# Stimuli-Responsive Luminophore Drives Mechanism Switch for Highly Efficient Electrochemiluminescence Immunosensing

**DOI:** 10.1021/jacs.5c10211

**Published:** 2025-08-26

**Authors:** Alessandro Fracassa, Giulia Ferrari, Maria Vittoria Balli, Isabella Rimoldi, Giorgio Facchetti, Lorenzo Arnal, Alessia Marconi, Matteo Calvaresi, Luca Prodi, Luisa De Cola, Giovanni Valenti

**Affiliations:** † Department of Chemistry “Giacomo Ciamician”, 201782 Alma Mater Studiorum − University of Bologna, Via Piero Gobetti 85, 40129 Bologna, Italy; ‡ Department of Pharmaceutical Science, DISFARM, 9304 University of Milan, Via Golgi 19, 20133 Milan, Italy; § IRCCS Azienda Ospedaliero-Universitaria di Bologna, via Albertoni 15, 40138 Bologna, Italy; ∥ Institute of Functional Interfaces, Karlsruhe Institute of Technology (KIT), 76344 Eggenstein-Leopoldshafen, Germany

## Abstract

Although widely used in clinical diagnostics, the sensitivity of electrochemiluminescence (ECL) bead-based immunoassays is intrinsically limited by the reaction mechanism driving the emission of [Ru­(bpy)_3_]^2+^ on the bead surface. Depending mostly on the coreactant oxidation, the ‘remote’ pathway is hindered by the slow coreactant oxidation rate and the short half-lives of electrogenerated radicals. In this work, we synthesized a [Ru­(bpy)_3_]^2+^ derivative featuring a stimuli-responsive disulfide bond in its linker to the bead. Electrogenerated tri-*n*-propylamine (TPrA) neutral radicals reduce disulfide moieties, electrochemically inducing the release of Ru­(II) labels in solution and thereby leading to an unprecedented mechanism shift toward the more efficient “homogeneous” ECL pathway. Leveraging ICP-MS, ECL microscopy, and finite element simulations, we demonstrate rapid bond cleavage and an impressive signal enhancement of up to 613%. Using an experimental configuration designed to emulate commercial clinical analysis, we developed an ECL-based immunoassay for the rapid detection of the SARS-CoV-2 Spike (S) protein in whole virus samples from swab formulations. The immunosensor incorporating the cleavable luminophore demonstrated a 40% lower detection limit and a 2-fold increase in sensitivity, while reducing TPrA consumption by 72%. These findings establish stimuli-responsive luminophores as a groundbreaking class of ECL labels, promising substantial improvements in the sensitivity of commercial biosensors.

## Graphical abstract

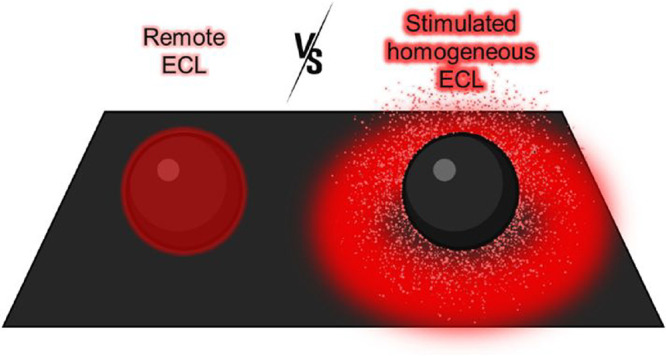

## Introduction

Diagnostic markers, also known as biomarkers, are biomolecules such as enzymes, proteins, peptides, and hormones, whose activity alterations are closely related to specific pathological conditions.^1^ As a result, biomarker quantification allows for an accurate prediction of disease progression and for an effective monitoring of the course of clinical treatments. This is why the exploration of noninvasive and sensitive quantification strategies is crucial for advancing analytical technologies that consistently measure clinically relevant analytes. In this context, the main challenge lies in the often exceptionally low concentration of biomarkers found in complex biological samples such as blood, urine, and tissues.

Electrochemiluminescence (ECL) is a phenomenon where an electrical stimulus triggers light emission at the electrode surface.^2^ As an electrochemical technique, ECL offers many intrinsic advantages over fluorescent or chemiluminescent biosensors, including precise control over both the position and timing of light emission as well as the absence of any external light source for excitation. These features collectively result in an outstanding signal-to-noise ratio and translate into the excellent analytical sensitivity that justifies the widespread use of ECL in clinical diagnostics.^3–5^ One of the most common types of ECL-based biosensors implies the construction of antibody sandwich assays, which capture and reveal analytes.^6^ In these systems, a capture antibody recognizes the target analyte within the sample, and then a transducer antibody – labeled with an ECL-active dye – binds to the captured analyte. This cascade of binding events creates a proportional relationship between the amount of captured analyte and the intensity of the ECL signal, allowing for biomarker quantification. ECL commercial assays usually exploit magnetic microbeads as a platform for binding the sandwich immunoassay.^7^ These immunobeads are first captured on the electrode surface by a magnet, and then, a photodetector collects the ECL signal generated by an emitter upon applying a potential. The most frequently used mechanism to achieve light emission is coreactant ECL. The coreactant is a sacrificial molecular species that undergoes a chemical transformation after being either oxidized or reduced. The typical bead-based immunoassay includes a luminophore, namely, tris­(2,2’-bipyridine)­ruthenium­(II) ([Ru­(bpy)_3_]^2+^), and tri*n*-propylamine (TPrA) as coreactant.

Despite its leading role as an electroanalytical technique, ECL still faces a few limitations. Notably, ECL is a surface-confined process that involves multiple steps to produce the final analytical signal, which is generally not stable.^8,9^ Although increasing the number of emitters enhances the ECL signal,^10–12^ this approach was demonstrated to yield diminishing returns, as the signal does not scale proportionally.^13^ The lack of a linear response is likely due to the quenching of the excited luminophores by neighboring oxidized or reduced species. Efforts to optimize the signal generation process are therefore particularly focused on developing novel luminophores exhibiting more intense and durable ECL emission.^14–17^

Stimuli-responsive materials have found widespread use across many fields;^18^ however, their application in the context of ECL remains significantly underexplored.^19,20^ In particular, stimuli-responsive ECL-based immunoassays have not yet been reported.

Here, we present an antithetical strategy to enhance the ECL intensity of a bead-based immunoassay. This approach aims to shift the paradigm of the research: from chasing a longer-lasting signal from the luminescent species to developing cleavable emitting labels. In the present work, we specifically focus on enhancing the analytical signal in bead-based ECL assays by swapping from the remote mechanism, adopted in systems including chemically inert labels (Figure 1a), to the more efficient homogeneous mechanism (see Results and Discussion section for mechanistic insights). In order to realize such a system, we designed and synthesized [Ru­(bpy)_2_(bpy-cys-NH_2_)]^2+^, a [Ru­(bpy)_3_]^2+^ derivative incorporating a disulfide moiety on the lateral chain of the ancillary ligand (Figure 1b). The disulfide bridge is purposely designed to be cleaved by electrogenerated coreactant radicals, thereby releasing the luminophore into the solution.

**Figure 1:**
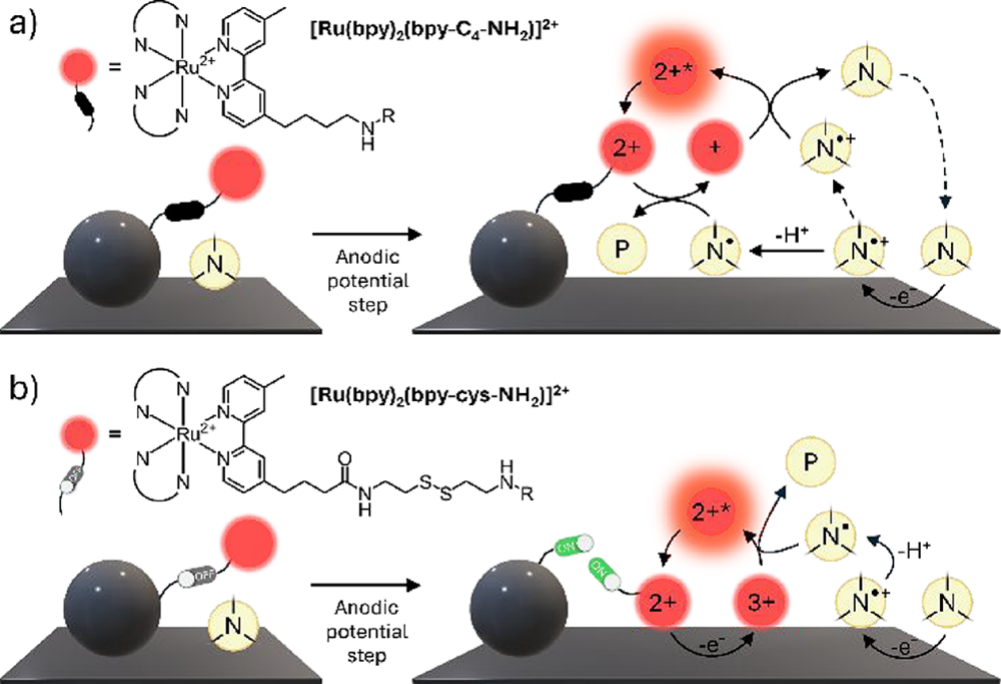
(a) Representation of a conventional bead-based immunoassay-like system in a TPrA solution (Ru­(C_4_)@Bead). Upon application of a suitable anodic potential, the traditional heterogeneous mechanism is activated, resulting in the ECL emission of the Ru­(II) derivative. (b) Representation of the cleavable bead-based immunoassay-like system in a TPrA solution (Ru­(S–S)@Bead). In its resting state, without any electrochemical stimulus, the disulfide bond is unresponsive to reduction, and its reactivity is switched off. Upon application of a suitable anodic potential for oxidizing TPrA, the reactivity of the disulfide bond is switched on by the reducing TPrA^·^ that cleave the linker, releasing the luminophore into the solution. If the applied potential is anodic enough to oxidize also the luminophore, then a more effective homogeneous ECL mechanism takes place. Red spheres labeled *2+*, *3+*, and *+* represent the different redox states of Ru­(II) complex involved in the ECL process, while 2+* denotes its emissive excited state. Yellow spheres labeled *N*, *N^•+^*, and *N^•^* correspond to tri-n-propylamine, its radical cation, and its neutral radical, respectively. For additional information see eqs 1–10.

This release activates the homogeneous ECL mechanism (Figure 1b) while simultaneously suppressing [Ru­(bpy)_3_]^2+^* quenching. We aim to explore the potential of this novel tool through an initial proof of concept study, followed by its application in a model bead-based assay designed to mimic the commercial version. For the mechanistic investigation, 2.8 μm magnetic beads decorated with carboxylic groups were covalently labeled with either one reference compound, namely, [Ru­(bpy)_2_(bpy-C_4_–NH_2_)]^2+^ (Ru­(C_4_)@Bead) or [Ru­(bpy)_2_(bpy-cys-NH_2_)]^2+^ (Ru­(S–S)@Bead).

By first leveraging the exceptional sensitivity of inductively coupled plasma mass spectrometry (ICP-MS) and then the remarkable spatial resolution of ECL microscopy, we demonstrate the feasibility of the proposed reaction mechanism under coreactant ECL working conditions. In particular, we compared the behavior of the cleavable Ru­(S–S)@Bead with the reference conventional Ru­(C_4_)@Bead system. The potential of this redox-responsive Ru­(II) label for early-stage clinical diagnostics was demonstrated through a bead-based immunoassay targeting the SARS-CoV-2 Spike (S) protein on intact viral envelopes derived from swab formulations using cheap and disposable carbon screen-printed electrodes (CSPE). The proposed approach, which closely replicates commercial immunoassay conditions, enables much faster analysis than current state-of-the-art techniques and offers enhanced sensitivity compared with the reference immunoassay, establishing cleavable labels as powerful transduction tools for next-generation ECL immunosensors.

## Results and Discussion

In conventional bead-based immunoassays, including those typically used in clinical analysis, [Ru­(bpy)_2_(bpy-C_4_–NH_2_)]^2+^ labels are tethered through an amide bond to the surface of microspheres, constraining the luminophores too far from the electrode surface to participate in any heterogeneous electron transfer process. Consequently, the ECL emission in this kind of system relies solely on the remote ECL mechanism, which is governed by the TPrA oxidation and the chemistry of its radicals. Upon sweeping the electrode to a potential sufficient to oxidize TPrA (*E*° = 0.83 V vs Ag/AgCl), the coreactant is converted to TPrA^·+^ (eq 2), which rapidly deprotonates to form the strongly reducing α-aminoalkyl radical TPrA^·^ (*E*° = −1.7 V vs Ag/AgCl, eq 3). The latter species then reduces [Ru­(bpy)_3_]^2+^ to [Ru­(bpy)_3_]^+^ (eq 4, where Im^+^ is the iminium ion, generated following the homogeneous TPrA^·^ oxidation), which is eventually oxidized by TPrA^·+^ to yield the luminescent [Ru­(bpy)_3_]^2+^* (eq 5).^21,22^

$${\mathrm{TPrAH}}^{+}⇆\mathrm{TPrA}+H^{+}$$ 1

$$\mathrm{TPrA}⇆{\mathrm{TPrA}}^{•+}+e^{-}$$ 2

$${\mathrm{TPrA}}^{•+}⇆{\mathrm{TPrA}}^{•}+H^{+}$$ 3

TPrA•+[Ru(bpy)3]2+⇆Im++[Ru(bpy)3]+ 4

$${\mathrm{TPrA}}^{•+}+[\mathrm{Ru}(\mathrm{bpy}{)}_{3}{]}^{+}⇆\mathrm{TPrA}+[\mathrm{Ru}(\mathrm{bpy}{)}_{3}{]}^{2+*}$$ 5

$$[\mathrm{Ru}(\mathrm{bpy}{)}_{3}{]}^{2+*}\to [\mathrm{Ru}(\mathrm{bpy}{)}_{3}{]}^{2+}+h\nu$$ 6

Despite being widely regarded as the most efficient coreactant for bead-based ECL assays, TPrA, together with intrinsic toxicity, presents major drawbacks that limit the overall effectiveness of the remote mechanism. The sluggish oxidation rate of TPrA indeed hinders the generation of radicals potentially available to react with the luminophore, thereby hampering its excitation rate.^23^ Although alternative amine coreactants exhibiting faster oxidation rates have been explored,^24,25^ their corresponding radical cations feature even shorter half-lives than TPrA^·+^, which itself is already limited to ∼200 μs. This brief lifespan acts as a bottleneck for the ECL emitting layer, by restricting its thickness to 2–3 μm from the electrode surface.^25–27^ Redox-mediated ECL,^28^ which has been extensively studied by our group and others in bead-based systems,^29–31^ similarly enhances coreactant oxidation through homogeneous catalysis, but does not significantly extend the emitting layer.

Replacing [Ru­(bpy)_2_(bpy-C_4_–NH_2_)]^2+^ with [Ru­(bpy)_2_(bpy-cys-NH_2_)]^2+^ introduces a redox-responsive disulfide moiety within the luminophore-bead linker, converting a conventionally stable bond into a labile bridge sensitive to reducing agents (see Supporting Information for full characterization and synthesis details). Several studies report that disulfide reduction to pairs of thiols in an aqueous environment is a two-electron event that follows an ECEC mechanism (*E* = electron transfer, *C* = chemical process).^32–35^ Upon the initial transfer of a single electron, a radical anion is formed, followed by immediate protonation in a first-order reaction with water. This protonation step facilitates the second reduction by lowering the required cathodic potential compared to the first one, thereby triggering a potential inversion. Finally, bond dissociation takes place following the uptake of the second electron and proton. During the ECL measurement, TPrA is oxidized to TPrA^·+^ (eq 2), and deprotonation leads to TPrA^·^ (eq 3). The latter radical, widely regarded as sufficiently long-lived to persist within the region occupied by the beads (i.e., at least ∼3 μm from the electrode surface), works as an electron donor in this breakable system where two equivalents of TPrA^·^ are required to homogeneously cleave the disulfide bridge (eq 7). In this way, the luminophore is no longer constricted solely to the surface of the conjugation platform. Instead, it can freely diffuse in solution, activating the homogeneous ECL mechanism if the electrode is swept to a sufficient anodic potential to oxidize both TPrA and [Ru­(bpy)_3_]^2+^ (*E*_1/2_(Ru^3+^/Ru^2+^) = 1.05 V vs Ag/AgCl). In this scenario, [Ru­(bpy)_3_]^3+^ is produced alongside coreactant radicals (eq 8) and replaces TPrA^·+^ as the main oxidizing agent as it is directly reduced by TPrA^·^, resulting in the excitation of the emitter, [Ru­(bpy)_3_]^2+^* (eq 9).

2TPrA•+Ru(S−S)@Bead+2H+⇆Im++[Ru(bpy)3]2+−S−H+Bead−S−H 7

$${[\mathrm{Ru}\left(\mathrm{bpy}{)}_{3}\right]}^{2+}\mathrm{-S-H}⇆{[\mathrm{Ru}\left(\mathrm{bpy}{)}_{3}\right]}^{3+}\mathrm{-S-H}+e^{-}$$ 8

[Ru(bpy)3]3+−S−H+TPrA•→[Ru(bpy)3]2+*−S−H+Im+ 9

$$[\mathrm{Ru}(\mathrm{bpy}{)}_{3}{]}^{2+*}\mathrm{-S-H}\to [\mathrm{Ru}(\mathrm{bpy}{)}_{3}{]}^{2+}\mathrm{-S-H}+h\nu$$ 10

The heterogeneous oxidation of the Ru­(II) complex is usually a fast process as it involves an outer-sphere electron transfer,^36^ unlike the coreactant, which undergoes a slower inner-sphere process that requires adsorption on the electrode surface.^24,37–39^ Additionally, the Ru^3+/2+^ couple is electrochemically fully reversible, making [Ru­(bpy)_3_]^3+^ a considerably more stable oxidizing agent compared to TPrA^·+^. These key differences translate to a dramatic improvement in the efficiency of ECL signal generation.

While Ru­(C_4_)@Bead and Ru­(S–S)@Bead systems effectively mimic the behavior of the conventional bead-based immunoassay, the covalent binding of the luminophores to the bead surface significantly increases the label loading on the microspheres compared to real biosensors.^8,9^ These features make covalently functionalized beads ideal candidates for proof-of-concept studies.

To assess the feasibility of the proposed [Ru­(bpy)_3_]^2+^ release from the cleavable Ru­(S–S)@Bead system and to quantify the amount of released Ru­(II) complex, we designed a ‘release experiment’ where we coupled electrochemistry to ICP-MS (Figure S1). The analysis aims to replicate ECL working conditions in a cell conveniently designed for retrieving the supernatant after multiple two-step chronoamperometry cycles at 1.4 V. In this potential region, TPrA oxidation is close to diffusion-controlled conditions at the glassy carbon (GC) electrode (Figure S2). Further details of the release experiment are provided in the Supporting Information. The Ru concentration in the release solution is determined to be 1.09 ± 0.07 g/L, supporting the expected cleavage of anchored luminophores under coreactant ECL working conditions.

To address the ECL behavior of labeled beads, we took advantage of ECL microscopy for a comparative study between Ru­(C_4_)@Bead and Ru­(S–S)@Bead. ECL microscopy integrates a potentiostat, which governs the electrochemistry of the investigated system, with an epifluorescence microscope for spatially resolving the ECL signal. This synergy has remodeled ECL into a powerful imaging technique that enables direct observation of objects that either initiate or influence ECL reactions, providing unparalleled mechanistic insights to shed light on ECL processes.^40,41^

ECL images of Ru­(C_4_)@Beads and Ru­(S–S)@Beads were collected with an EM-CCD camera by integrating the emission during a two-step chronoamperometry measurement on a GC electrode (Figure 2). The applied potential sequence consisted of an initial step at 0 V followed by a second step of 2.5 V (Figure S3). Ru­(C_4_)@Bead manifests the common ECL behavior, with an emission strictly confined to the bead surface and a negligible background signal (Figure 2a). Any deviation from this typical pattern in Ru­(S–S)@Bead can, therefore, be attributed to the homogeneous ECL following the cleavage of disulfide bridges. As evident in Figure 2b, Ru­(S–S)@Bead displays an intense background signal throughout the entire image, which is reflected in the shape of the ECL profiles (Figure 2c). Details about ECL profile elaboration are provided in the Supporting Information (Figure S4). Considering that the region implied by the peak corresponds to the bead surface, while the remainder represents the surrounding environment, Ru­(S–S)@Bead solution emission (∼6900 a.u., Figure 2c, red line) exhibits 208-fold stronger intensity compared to the negligible signal observed for Ru­(C_4_)@Bead (∼30 a.u., Figure 2c, gray line).

**Figure 2:**
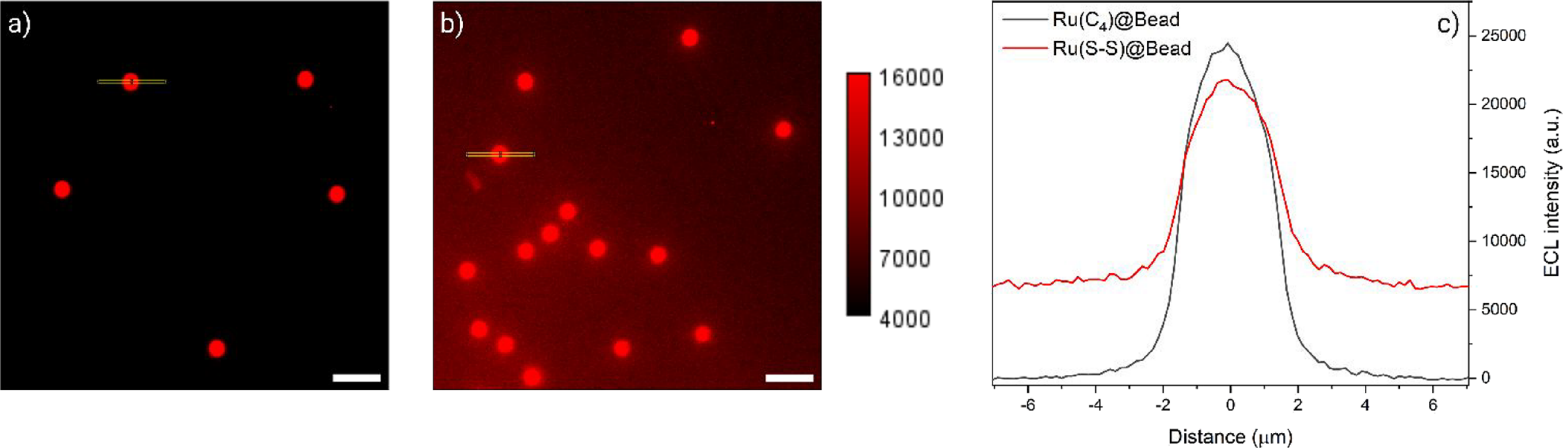
ECL images of (a) Ru­(C_4_)@Bead and (b) Ru­(S–S)@Bead in 0.3 M PB with 180 mM TPrA (pH 6.8). The images were captured with an EM-CCD camera by recording the ECL signal for 45 s during a two-step chronoamperometry measurement: 2 s at 0 V versus Ag/AgCl and 43 s at 2.5 V vs Ag/AgCl. Magnification, ×100; objective numerical aperture, 1.1; gain, 1; sensitivity, 250; contrast scale, 4000–16,000; scale bar, 10 μm. The yellow rectangles (14.28 × 0.63 μm) centered on the beads represent the regions of interest (ROI) used to extract ECL intensity profiles along the radial direction. A red lookup table was applied to the native greyscale images in (a) and (b) to generate false-color images resembling the emission of [Ru­(bpy)_3_]^2+^. (c) Comparison between the single-bead ECL intensity profiles of Ru­(C_4_)@Bead (gray line) and Ru­(S–S)@Bead (red line). Data are averaged over a minimum of five beads (*n* ≥ 5).

Further supporting the cleavage of Ru­(II) labels, the solution emission of Ru­(S–S)@Bead shows a clear gradient, increasing in intensity as the beads come closer together (Figure 2b). This gradient can be attributed to a higher bead density in a certain spatial region that translates to a higher concentration of released ruthenium and, in turn, to a stronger ECL emission. Furthermore, ECL profiles hint at a weaker emission from the surface of beads (i.e., peak intensity) labeled with the cleavable complex compared to its nonbreakable counterpart. Assuming an equal degree of functionalization in both cases, this behavior can be attributed to the release of luminophores in Ru­(S–S)@Bead, which amplifies the background signal at the expense of the ECL emission arising from the beads themselves. Overall, the whole luminescence intensity is increased, in agreement with the higher efficiency of the homogeneous mechanism, confirming the idea on which this work is based.

To monitor the kinetics of disulfide bond cleavage, ECL images of labeled beads on a GC electrode were captured at 500 ms intervals during the same two-step chronoamperometry measurement previously employed (Figure S5a). Details of ECL elaboration can be found in the Supporting Information. Notably, Ru­(S–S)@Bead consistently exhibits a stronger ECL intensity throughout the measurement compared to Ru­(C_4_)@Bead, with the enhancement being most pronounced immediately after the anodic potential application (Figure S5b). This behavior can be explained by Figure 3a,b, representing the first frame of emission of Ru­(C_4_)@Bead and Ru­(S–S)@Bead, respectively, captured after application of the anodic potential. The background emission of Ru­(C_4_)@Bead is not significant, whereas Ru­(S–S)@Bead readily displays intense homogeneous ECL, suggesting that most of the disulfide bonds are cleaved within the first hundreds of milliseconds. This behavior was rationalized using finite element simulations, Figure 3c, where the green-to-blue scale is the Ru­(II) label distribution on the beads and the black-to-yellow scale is the spatial distribution of *h*ν emitted (Figure 3c and ESI for full details). Specifically, we modeled the limiting case involving only the cleavage of bounded Ru­(II) from the bead and its homogeneous emission, while neglecting any other reactions occurring at the bead surface. In line with our mechanism (eqs 7–10), ECL emission occurs from the released Ru­(II) complex (black-to-yellow scale) while the concentration of Ru­(II) bound on the bead gradually decreases (green-to-blue scale) from the electrode surface to the top of the microsphere according to the TPrA^·^ inward flux (eq 3, Figure S6).^9^ The ECL behavior is well reproduced in finite element simulations using a second-order rate constant for the cleavage reaction (*k*_SS_) as low as 5 × 10^4^ M^–1^ s^–1^. However, a higher value is likely more realistic considering that, in the actual system, disulfide bond cleavage kinetically competes with the reduction of Ru^2^^+^ by TPrA^·^, a process with a reported rate constant ranging from 4.2 × 10^5^ to 6 × 10^6^ M^–1^ s^–1^.^25^ A slightly higher value for *k*_SS_ would also align with literature values for either the homogeneous reduction or oxidation of disulfide bonds.^42–44^ Although one might assume Ru­(II) to build up over time with increasing ECL until reaching a plateau, Ru­(S–S)@Bead signal stability, after the initial peak, decays faster than both Ru­(C_4_)@Bead (Figure S8) and solutions including TPrA and freely diffusing [Ru­(bpy)_3_]^2+^ (Figure S9), where the luminophore concentration is comparable to that in Ru­(S–S)@Bead after cleavage (as high as 30 nM, see Supporting Information). ECL signal stability in homogeneous systems is typically attributed to the depletion of electroactive species such as [Ru­(bpy)_3_]^2+^ and TPrA; thus, deviations observed in Ru­(C_4_)@Bead and Ru­(S–S)@Bead suggest that additional factors significantly influence emission behavior.

**Figure 3:**
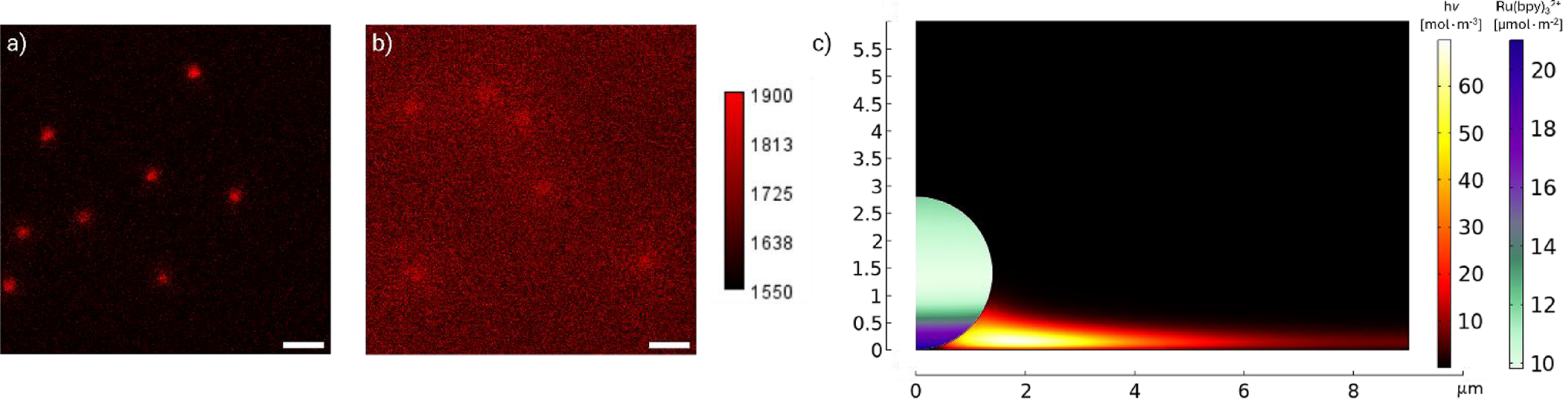
ECL images of (a) Ru­(C_4_)@Bead and (b) Ru­(S–S)@Bead in 0.3 M PB with 180 mM TPrA (pH 6.8). The images represent the 5th frame (i.e., image captured 500 ms after the anodic potential sweep) of an ECL decay measurement recorded with an EM-CCD camera by capturing a frame every 500 ms over a total duration of 13 s during a two-step chronoamperometry measurement: 2 s at 0 V vs Ag/AgCl and 11 s at 2.5 V vs Ag/AgCl. Magnification, ×100; objective numerical aperture, 1.1; gain, 1; sensitivity, 250; contrast scale, 1550–1900; scale bar, 10 μm. (c) Simulated spatial distribution of Ru­(II) labels on the bead surface at 500 ms during the anodic potential sweep (in μmol m^–2^, green-to-blue scale on the right) and spatial distribution of *h*ν emitted from cleaved [Ru­(bpy)_3_]^2+^* and integrated over 500 ms (in mol m^–3^, black-to-yellow scale on the left) when *k*_SS_ is 1 × 10^5^ M^–1^ s^–1^.

In Ru­(C_4_)@Bead, anchoring [Ru­(bpy)_3_]^2+^ seems to stabilize the signal by confining the luminophore to the microspheres, thereby neglecting Ru­(II) mass transport limitations. On the other hand, in Ru­(S–S)@Bead, the diffusion of cleaved ECL labels from the bead's surroundings to the bulk solution provides an additional pathway to accelerate emission decay by progressive [Ru­(bpy)_3_]^2+^ dilution. Commercial bead-based immunoassays usually rely on high concentrations of TPrA, typically 180 mM, to compensate for its slow oxidation rate at the electrode surface. On the other hand, homogeneous ECL systems generally perform best with more diluted coreactant solutions.^45–48^ As we demonstrated, the Ru­(S–S)@Bead system bridges this gap by adopting both heterogeneous and homogeneous mechanisms to generate the ECL signal. In light of this, believing that it is worthwhile to investigate the impact of TPrA concentration, we collected ECL images of both Ru­(C_4_)@Bead and Ru­(S–S)@Bead on a GC electrode in the presence of 50, 100, or 180 mM of TPrA (Figure S10–S12). The emission was triggered by a two-step chronoamperometry measurement, as previously described. The ECL signal was integrated as reported in the Supporting Information, and the resulting values are plotted as a function of the coreactant concentration (Figure 4). Ru­(C_4_)@Bead and Ru­(S–S)@Bead exhibit two diametrically opposed trends with a decreasing TPrA concentration. While the ECL intensity of the conventional system tends to weaken as the coreactant concentration drops, the breakable system shows a massive surge in emission. In particular, the ECL signal of Ru­(C_4_)@Bead remains mostly unchanged from 180 to 100 mM TPrA, and eventually plunges at 50 mM. This behavior is not surprising, as heterogeneous systems require high concentrations of TPrA to overcome the bottleneck caused by its sluggish oxidation rate. Thus, at 50 mM, Ru­(C_4_)@Bead might lack TPrA^·+^ radicals to express a strong ECL signal. The ECL signal of Ru­(S–S)@Bead, instead, steadily grows brighter across the entire concentration range, from 180 to 50 mM TPrA. Overall, at their best conditions, Ru­(S–S)@Beads (TPrA 50 mM) offers a 3.6-fold signal increase with respect to Ru­(C_4_)@Beads (TPrA 100 mM).

**Figure 4:**
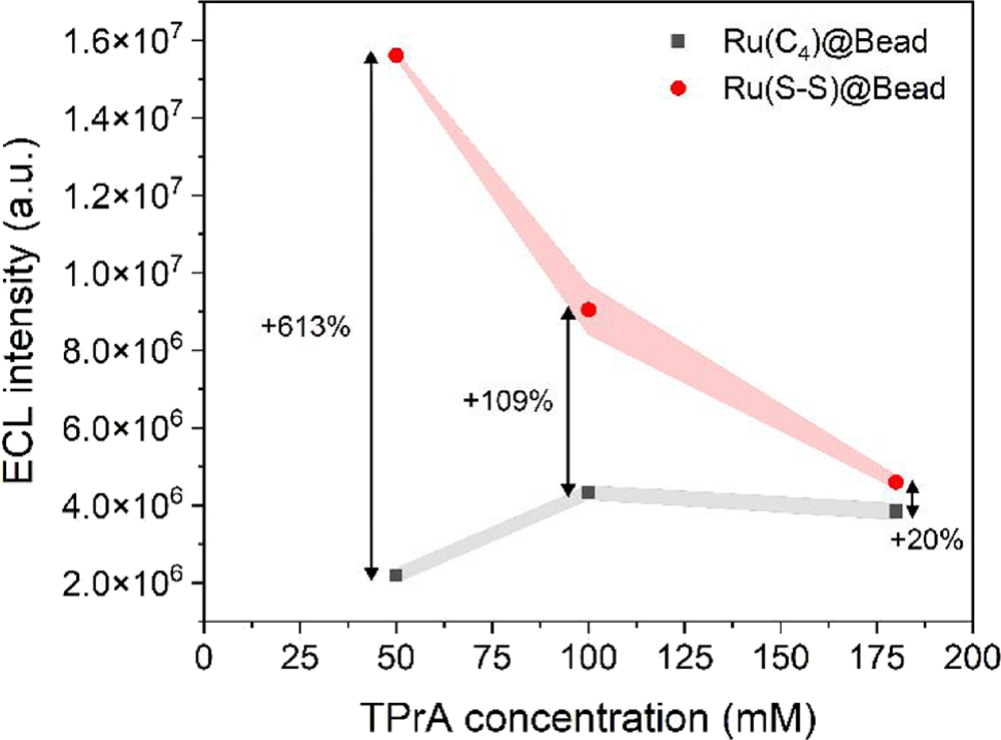
Effect of TPrA concentration on the ECL intensity of Ru­(C_4_)@Bead (gray line) and Ru­(S–S)@Bead (red line) on GC. ECL intensities were determined from integrated ECL images, by integrating the signal over a ROI centered on the beads and averaging the results over a minimum of five beads (*n* ≥ 5). Shaded bands represent the standard error.

Although the mechanism swap still occurs on a Pt electrode (Figure S13), the trend observed on GC upon changing TPrA concentration does not appear to be reproducible on the alternative surface (Figure S14), suggesting that it is not a general property of this class of breakable compounds.

Analysis of the ECL profiles derived from integrated images (Figure S15) offers valuable insights into the behavior of Ru­(S–S)@Bead. The trend observed in Figure 4 remains consistent whether the plotted values include both the bead and the surrounding solution (as in Figure 4) or focus solely on the homogeneous ECL intensity (i.e., value at the edge of the ECL profile) (Figure S16). Furthermore, the intensity of the homogeneous ECL in solution progressively strengthens with a decreasing TPrA concentration. These observations point to two key conclusions: first, the observed trend is likely governed by the homogeneous mechanism; and second, the latter reaction pathway becomes increasingly dominant at lower TPrA concentrations. In this regard, a similar ECL-[TPrA] trend has already been observed in a work from Xu and co-workers where they recorded the homogeneous ECL emission of [Ru­(bpy)_3_]^2+^ on a GC electrode in coreactant solutions with different TPrA concentrations.^48^ They reported that the ECL intensity of the [Ru­(bpy)_3_]^2+^/TPrA system increases with increasing coreactant concentration, achieving the brightest intensity with a 40 mM TPrA solution. Beyond this concentration, the emission intensity progressively drops. Since a similar behavior was observed with the experimental apparatus employed throughout this work (Figure S17), it is reasonable to assume that the inverse relationship between the ECL intensity of Ru­(S–S)@Bead and the TPrA concentration is defined by the effectiveness of the homogeneous mechanism, which generates the strongest emission in solutions with 40/50 mM of coreactant. However, one must also consider that – while the homogeneous ECL increases as TPrA concentration decreases – the opposite trend occurs for the emission from Ru­(II) labels bound to the beads (Figure S18). This signal, reflected in the difference between the profile peak and the profile edge, steadily decreases as TPrA is diluted from 180 to 50 mM. This phenomenon may suggest a more efficient release of Ru­(II) labels at lower coreactant concentrations, but further investigation is needed to confirm this hypothesis.

Early-stage clinical diagnostics are essential to preventing irreversible health outcomes such as cancer progression or the uncontrolled spread of infectious diseases. In particular, the rapid and sensitive detection of viral infections—especially in asymptomatic individuals—is critical for effective epidemic management, as highlighted during the COVID-19 (COrona VIrus Disease 2019) pandemic.

The versatility of the redox-responsive Ru­(II) label approach in bioanalysis is therefore exploited in a bead-based immunoassay targeting SARS-CoV-2 spike protein subunit 1 (S1). The assay detects the antigen directly on the surface of whole virions within a complex matrix, such as nasopharyngeal swab samples (Figures 5a and S19).^49^ The ECL signal enhancement is compared to the noncleavable Ru­(II) luminophore employing an experimental setup affine to commercial bead-based immunoassays, while also implementing cheap and easy-to-handle disposable CSPE and diluting coreactant to 50 mM TPrA (see Figure 5b and Supporting Information for the detailed description of the immunoassay). To optimize signal generation, we tailored the lateral chain length of the ancillary ligand (Supporting Information) to minimize steric hindrance between the luminophore and antibody.

**Figure 5:**
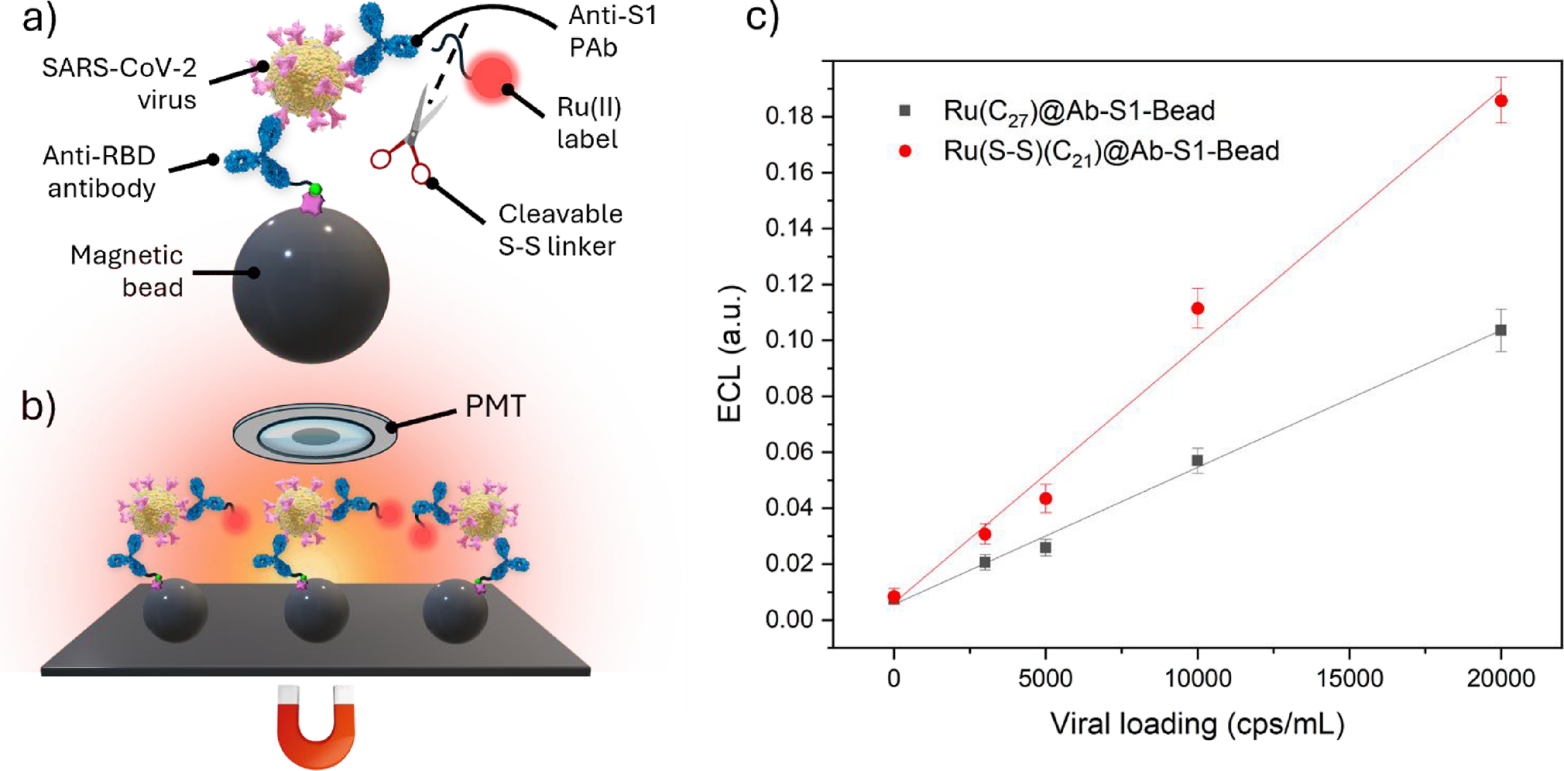
(a) Immunoassay structure of either Ru­(C_27_)@Ab-S1-Bead or Ru­(S–S)­(C_21_)@Ab-S1-Bead. Immunoassays were prepared on Anti-RBD–functionalized magnetic beads by simultaneously adding SARS-CoV-2 and Ru­(II)-labeled detection antibodies. (b) Experimental setup for collective beads measurements where beads are collected with a magnet under the working electrode and the ECL is acquired with a photomultiplier tube. (c) Calibration curves for Ru­(C_27_)@Ab-S1-Bead (gray squares) and Ru­(S–S)­(C_21_)@Ab-S1-Bead (red circles) upon decreasing the viral loading of whole SARS-CoV-2 virus from 20,000 to 0 cps mL^–1^. The plotted ECL values were determined by integrating the *ECL-time* curves reported in the Supporting Information (Figure S19). Each data point corresponds to the average of three independent replicates (*n* = 3), with error bars indicating the standard deviation.

The proposed immunoassay is supported on 2 μm streptavidin-coated magnetic beads functionalized with biotinylated anti-RBD antibodies. Following incubation with varying viral load, virions are sandwiched between the beads’ surface and the detection antibodies (Figure 5a), labeled with either the reference (Ru­(C_27_)@Ab-S1-Bead) or the breakable luminophore (Ru­(S–S)­(C_21_)@Ab-S1-Bead). In this case, all of the photons generated at the electrode surface are collected without spatial resolution with a PMT (Figure 5b). Comparing the calibration curves of Ru­(S–S)­(C_21_)@Ab-S1-Bead and Ru­(C_27_)@Ab-S1-Bead reveals a higher ECL signal across all antibody concentrations when the beads are labeled with the breakable Ru­(II) complex (Figures 5c and S20). This behavior results in a 2-fold increase in sensitivity within a clinically relevant concentration range for early diagnosis,^50^ reducing the likelihood of either false positive or false negative. At the same time, the use of cleavable labels lowers the limit of detection by 40%, from 1700 to 1100 cps mL^–1^. Beyond enhancing the analytical performance of the bead-based immunoassay, the proposed approach significantly reduces the overall analysis time (Table S1) and improves coreactant economy by reducing consumption of toxic TPrA by 72% compared to standard commercial immunosensors.

## Conclusions

In this study, we presented the synthesis and ECL characterization of a novel [Ru­(bpy)_3_]^2+^ derivative featuring a stimuli-responsive disulfide in the conjugation functional group. Magnetic beads labeled with this complex exhibit a unique pattern of emission during anodic potential sweeps in a TPrA solution. Extensive investigations combining ICP-MS, ECL microscopy, and finite element simulations unraveled a mechanism involving a very fast disulfide bond cleavage upon reaction with reducing TPrA radicals, thereby releasing the luminophore into the solution. Freely diffusing Ru­(II) enables its direct oxidation, driving a transition from the conventional remote ECL pathway to a more efficient homogeneous mechanism. Compared to beads labeled with standard [Ru­(bpy)_3_]^2+^, this shift leads to a remarkable signal enhancement up to 613%. This breakable luminophore is eventually proposed as a detection label within a bead-based immunoassay developed to target the SARS-CoV-2 S1 protein on the whole virus. Replacing conventional [Ru­(bpy)_3_]^2+^ with the redox-responsive derivative doubles the sensitivity and improves the detection limit by 40% in diluted TPrA solution, showcasing the superior efficiency of the homogeneous ECL mechanism in bead-based immunosensing. Although complete cleavage of Ru­(II) from the surface of Ru­(S–S)@Bead has not yet been achieved, different stimuli-responsive ligands are currently under investigation in our laboratories. Nonetheless, this strategy introduces stimuli-responsive luminophores as a promising new class of ECL immunolabels, establishing their controlled instability as a key parameter to drive a mechanism switch, opening new avenues for significant performance gains.

## Supplementary Material



## Data Availability

The data presented in Figures 2–4, S7, S14, S15 are openly available in AMS Acta at 10.6092/unibo/amsacta/8318. Figure 5 is openly available in AMS Acta at 10.6092/unibo/amsacta/8482.

## Notes

Synthesis and characterization of coordination complexes, chemicals, instrumentation, and experimental details, raw ECL images processing methods, ECL signal stability measurements, COMSOL simulation model, single beads ECL images, and collective beads ECL profiles (PDF)

## ⊥

Department of Chemistry, Maynooth University, W23 F2H6 Maynooth, Ireland

## References

[1] Ahmad A., Imran M., Ahsan H. (2023). Biomarkers as Biomedical Bioindicators:
Approaches and Techniques for the Detection, Analysis, and Validation
of Novel Biomarkers of Diseases. Pharmaceutics.

[2] Giagu G., Fracassa A., Fiorani A., Villani E., Paolucci F., Valenti G., Zanut A. (2024). From Theory
to Practice: Understanding
the Challenges in the Implementation of Electrogenerated Chemiluminescence
for Analytical Applications. Microchim. Acta.

[3] Sobhanie E., Salehnia F., Xu G., Hamidipanah Y., Arshian S., Firoozbakhtian A., Hosseini M., Ganjali M. R., Hanif S. (2022). Recent Trends and Advancements in Electrochemiluminescence Biosensors
for Human Virus Detection. TrAC - Trends Anal.
Chem..

[4] Barhoum A., Altintas Z., Devi K. S. S., Forster R. J. (2023). Electrochemiluminescence
Biosensors for Detection of Cancer Biomarkers in Biofluids: Principles,
Opportunities, and Challenges. Nano Today.

[5] Guo, W. ; Ding, H. ; Su, B. Electrochemiluminescence for Biomolecule Analysis. In Encyclopedia of Analytical Chemistry; John Wiley & Sons, Ltd, 2024; 1–23.

[6] Yu J., Stankovic D., Vidic J., Sojic N. (2024). Recent Advances in
Electrochemiluminescence Immunosensing. Sensors
& Diagnostics.

[7] Faatz, E. ; Finke, A. ; Josel, H. P. ; Prencipe, G. ; Quint, S. ; Windfuhr, M. Chapter 15: Automated Immunoassays for the Detection of Biomarkers in Body Fluids. In Analytical Electrogenerated Chemiluminescence; Royal Society of Chemistry, 2019; 443–470.

[8] Dutta P., Han D., Goudeau B., Jiang D., Fang D., Sojic N. (2020). Reactivity
Mapping of Luminescence in Space: Insights into Heterogeneous Electrochemiluminescence
Bioassays. Biosens. Bioelectron..

[9] Han D., Fang D., Valenti G., Paolucci F., Kanoufi F., Jiang D., Sojic N. (2023). Dynamic Mapping
of Electrochemiluminescence
Reactivity in Space: Application to Bead-Based Assays. Anal. Chem..

[10] Kesarkar S., Valente S., Zanut A., Palomba F., Fiorani A., Marcaccio M., Rampazzo E., Valenti G., Paolucci F., Prodi L. (2019). Neutral Dye-Doped
Silica Nanoparticles for Electrogenerated Chemiluminescence
Signal Amplification. J. Phys. Chem. C.

[11] Han D., Goudeau B., Lapeyre V., Ravaine V., Jiang D., Fang D., Sojic N. (2022). Enhanced Electrochemiluminescence
at Microgel-Functionalized Beads. Biosens. Bioelectron..

[12] Fu W., Wang X., Ying X., Sun T., Wang Y., Wang J., Su B. (2024). Electrochemiluminescence
Lateral
Flow Immunoassay Using Ruthenium­(II) Complex-Loaded Dendritic Mesoporous
Silica Nanospheres for Highly Sensitive and Quantitative Detection
of SARS-CoV-2 Nucleocapsid Protein. Adv. Funct.
Mater..

[13] Valenti G., Rampazzo E., Bonacchi S., Petrizza L., Marcaccio M., Montalti M., Prodi L., Paolucci F. (2016). Variable Doping
Induces
Mechanism Swapping in Electrogenerated Chemiluminescence of Ru­(Bpy)­32+
Core-Shell Silica Nanoparticles. J. Am. Chem.
Soc..

[14] Yu L., Liu Y., Zhou M. (2016). Improved Electrochemiluminescence Labels for Heterogeneous
Microbead Immunoassay. Anal. Bioanal. Chem..

[15] Chen L., Hayne D. J., Doeven E. H., Agugiaro J., Wilson D. J. D., Henderson L. C., Connell T. U., Nai Y. H., Alexander R., Carrara S., Hogan C. F., Donnelly P. S., Francis P. S. (2019). A Conceptual
Framework for the Development of Iridium­(Iii) Complex-Based Electrogenerated
Chemiluminescence Labels. Chem. Sci..

[16] Cao Z., Shu Y., Qin H., Su B., Peng X. (2020). Quantum Dots with Highly
Efficient, Stable, and Multicolor Electrochemiluminescence. ACS Cent. Sci..

[17] Wang Z.-X., Liu K.-Q., Li F., Li H.-Y., Wang W., Gao H. (2024). Long-Term Stable Electrochemiluminescence
of Perovskite Quantum Dots
in Aqueous Media. Chem. Commun..

[18] Zhang X., Chen L., Lim K. H., Gonuguntla S., Lim K. W., Pranantyo D., Yong W. P., Yam W. J. T., Low Z., Teo W. J., Nien H. P., Loh Q. W., Soh S. (2019). The Pathway to Intelligence:
Using Stimuli-Responsive Materials as
Building Blocks for Constructing Smart and Functional Systems. Adv. Mater..

[19] Ben
Trad F., Wieczny V., Delacotte J., Morel M., Guille-Collignon M., Arbault S., Lemaître F., Sojic N., Labbé E., Buriez O. (2022). Dynamic Electrochemiluminescence Imaging of Single
Giant Liposome Opening at Polarized Electrodes. Anal. Chem..

[20] Li B., Lu Y., Huang X., Sojic N., Jiang D., Liu B. (2025). Stimuli-Responsive
DNA Nanomachines for Intracellular Targeted Electrochemiluminescence
Imaging in Single Cells. Angew. Chem., -Int.
Ed..

[21] Miao W., Choi J. P., Bard A. J. (2002). Electrogenerated Chemiluminescence
69: The Tris­(2,2′-Bipyridine)­Ruthenium­(II), (Ru­(Bpy)­32+)/Tri-n-Propylamine
(TPrA) System Revisited - A New Route Involving TPrA.+ Cation Radicals. J. Am. Chem. Soc..

[22] Zanut A., Fiorani A., Canola S., Saito T., Ziebart N., Rapino S., Rebeccani S., Barbon A., Irie T., Josel H. P., Negri F., Marcaccio M., Windfuhr M., Imai K., Valenti G., Paolucci F. (2020). Insights into
the Mechanism of Coreactant Electrochemiluminescence Facilitating
Enhanced Bioanalytical Performance. Nat. Commun..

[23] Fracassa A., Mariani C., Fiorani A., Einaga Y., Hogan C. F., Paolucci F., Sojic N., Francis P. S., Valenti G. (2025). Overcoming
Kinetic Barriers of Remote Electrochemiluminescence on Boron-Doped
Diamond via Catalytic Coreactant Oxidation. Chem. Commun..

[24] Wang Y., Ding J., Zhou P., Liu J., Qiao Z., Yu K., Jiang J., Su B. (2023). Electrochemiluminescence
Distance
and Reactivity of Coreactants Determine the Sensitivity of Bead-Based
Immunoassays. Angew. Chem., -Int. Ed..

[25] Feng Y., Wang C., Zhou W., Yang X., Paolucci F., Valenti G., Qi H. (2025). Tomography
Electrogenerated Chemiluminescence
Imaging from Magnetic Microbeads. Small.

[26] Sentic M., Milutinovic M., Kanoufi F., Manojlovic D., Arbault S., Sojic N. (2014). Mapping Electrogenerated Chemiluminescence
Reactivity in Space: Mechanistic Insight into Model Systems Used in
Immunoassays. Chem. Sci..

[27] Feng Y., Zhou W., Wang X., Zhang J., Zou M., Zhang C., Qi H. (2023). Imaging and
Simulation of Ruthenium
Derivative Coating Microbeads at the Opaque Electrode with Electrogenerated
Chemiluminescence. Chem. Biomed. Imaging.

[28] Kerr E., Hayne D. J., Soulsby L. C., Bawden J. C., Blom S. J., Doeven E. H., Henderson L. C., Hogan C. F., Francis P. S. (2022). A Redox-Mediator
Pathway for Enhanced Multi-Colour Electrochemiluminescence in Aqueous
Solution. Chem. Sci..

[29] Kerr E., Knezevic S., Francis P. S., Hogan C. F., Valenti G., Paolucci F., Kanoufi F., Sojic N. (2023). Electrochemiluminescence
Amplification in Bead-Based Assays Induced by a Freely Diffusing Iridium­(III)
Complex. ACS Sensors.

[30] Fracassa A., Santo C. I., Kerr E., Knežević S., Hayne D. J., Francis P. S., Kanoufi F., Sojic N., Paolucci F., Valenti G. (2024). Redox-Mediated
Electrochemiluminescence
Enhancement for Bead-Based Immunoassay. Chem.
Sci..

[31] Adamson N. S., Blom S. J., Doeven E. H., Connell T. U., Hadden C., Knežević S., Sojic N., Fracassa A., Valenti G., Paolucci F., Ding J., Wang Y., Su B., Hua C., Francis P. S. (2024). Electrochemiluminescence Enhanced
by a Non-Emissive Dual Redox Mediator. Angew.
Chem., Int. Ed..

[32] Benniston A. C., Allen B. D., Harriman A., Llarena I., Rostron J. P., Stewart B. (2009). Accessing Molecular Memory via a Disulfide Switch. New J. Chem..

[33] Cattaneo M., Schiewer C. E., Schober A., Dechert S., Siewert I., Meyer F. (2018). 2,2′-Bipyridine Equipped with a Disulfide/Dithiol Switch for
Coupled Two-Electron and Two-Proton Transfer. Chem. - A Eur. J..

[34] Hua S. A., Cattaneo M., Oelschlegel M., Heindl M., Schmid L., Dechert S., Wenger O. S., Siewert I., González L., Meyer F. (2020). Electrochemical and
Photophysical Properties of Ruthenium­(II) Complexes
Equipped with Sulfurated Bipyridine Ligands. Inorg. Chem..

[35] Ould
Mohamed L., Abtouche S., Ghoualem Z., Assfeld X. (2024). Unraveling
Redox Pathways of the Disulfide Bond in Dimethyl Disulfide: Ab Initio
Modeling. J. Mol. Model..

[36] Shen M., Bard A. J. (2011). Localized Electron Transfer and the Effect of Tunneling
on the Rates of Ru­(Bpy)­32+ Oxidation and Reduction as Measured by
Scanning Electrochemical Microscopy. J. Am.
Chem. Soc..

[37] Zu Y., Bard A. J. (2001). Electrogenerated Chemiluminescence. 67. Dependence
of Light Emission of the Tris­(2,2′)­Bipyridylruthenium­(II)/Tripropylamine
System on Electrode Surface Hydrophobicity. Anal. Chem..

[38] Valenti G., Fiorani A., Li H., Sojic N., Paolucci F. (2016). Essential
Role of Electrode Materials in Electrochemiluminescence Applications. ChemElectroChem.

[39] Sakanoue K., Fiorani A., Santo C. I., Irkham, Valenti G., Paolucci F., Einaga Y. (2022). Boron-Doped Diamond Electrode Outperforms the State-of-the-Art
Electrochemiluminescence from Microbeads Immunoassay. ACS Sensors.

[40] Knežević S., Han D., Liu B., Jiang D., Sojic N. (2024). Electrochemiluminescence
Microscopy. Angew. Chem., Int. Ed..

[41] Xing Z., Lu X., Zhang Z., Zhao Y., Cao Y., Zhou Y., Zhu J. J. (2025). Electrochemiluminescence Microscopy
in Nano-Electrochemistry
Research: Unraveling the Underlying Principles, Tracing the Evolutionary
Developments, and Charting the Prospective Trajectories. Adv. Funct. Mater..

[42] Hoffman M. Z., Hayon E. (1972). One-Electron Reduction
of the Disulfide Linkage in Aqueous Solution.
Formation, Protonation and Decay Kinetics of the RSSR- Radical. J. Am. Chem. Soc..

[43] Karimi M., Ignasiak M. T., Chan B., Croft A. K., Radom L., Schiesser C. H., Pattison D. I., Davies M. J. (2016). Reactivity of Disulfide
Bonds Is Markedly Affected by Structure and Environment: Implications
for Protein Modification and Stability. Sci.
Rep..

[44] Ruccolo S., Emmert M., Bottecchia C., Qin Y., Barrientos R., Raymond K., Haley M. (2024). Electrocatalytic Reduction
of Disulfide
Bonds across Chemical Modalities. Org. Lett..

[45] Liu X., Shi L., Niu W., Li H., Xu G. (2007). Environmentally Friendly
and Highly Sensitive Ruthenium­(II) Tris­(2,2′-Bipyridyl) Electrochemiluminescent
System Using 2-(Dibutylamino)­Ethanol as Co-Reactant. Angew. Chemie Int. Ed..

[46] Han S., Niu W., Li H., Hu L., Yuan Y., Xu G. (2010). Effect of
Hydroxyl and Amino Groups on Electrochemiluminescence Activity of
Tertiary Amines at Low Tris­(2,2′-Bipyridyl)­Ruthenium­(II) Concentrations. Talanta.

[47] Kitte S. A., Wang C., Li S., Zholudov Y., Qi L., Li J., Xu G. (2016). Electrogenerated
Chemiluminescence of Tris­(2,2’-Bipyridine)­Ruthenium­(II)
Using N-(3-Aminopropyl)­Diethanolamine as Coreactant. Anal. Bioanal. Chem..

[48] Parveen S., Chen Y., Yuan Y., Hu L., Zhang W., Gilani M. R. H. S., Shi Y., Aziz-ur-Rehman, Xu G. (2021). Electrochemiluminescence
of [Ru­(Bpy)­3]­2+/Tripropylamine at Glassy Carbon, Platinum, and Palladium
Electrodes. Sensors and Actuators Reports.

[49] Fabiani L., Saroglia M., Galatà G., De Santis R., Fillo S., Luca V., Faggioni G., D’Amore N., Regalbuto E., Salvatori P., Terova G., Moscone D., Lista F., Arduini F. (2021). Magnetic Beads
Combined with Carbon
Black-Based Screen-Printed Electrodes for COVID-19: A Reliable and
Miniaturized Electrochemical Immunosensor for SARS-CoV-2 Detection
in Saliva. Biosens. Bioelectron..

[50] Wyllie A. L., Fournier J., Casanovas-Massana A., Campbell M., Tokuyama M., Vijayakumar P., Warren J. L., Geng B., Muenker M. C., Moore A. J., Vogels C. B. F., Petrone M. E., Ott I. M., Lu P., Venkataraman A., Lu-Culligan A., Klein J., Earnest R., Simonov M., Datta R., Handoko R., Naushad N., Sewanan L. R., Valdez J., White E. B., Lapidus S., Kalinich C. C., Jiang X., Kim D. J., Kudo E., Linehan M., Mao T., Moriyama M., Oh J. E., Park A., Silva J., Song E., Takahashi T., Taura M., Weizman O.-E., Wong P., Yang Y., Bermejo S., Odio C. D., Omer S. B., Dela
Cruz C. S., Farhadian S., Martinello R. A., Iwasaki A., Grubaugh N. D., Ko A. I. (2020). Saliva or Nasopharyngeal
Swab Specimens for Detection of SARS-CoV-2. N. Engl. J. Med..

