# Supplementary material for: Stimuli-Responsive Luminophore Drives Mechanism Switch for Highly Efficient Electrochemiluminescence Immunosensing
Source: J Am Chem Soc. 2025 Aug 26;147(39):35501–9. doi: 10.1021/jacs.5c10211 (PMC12498414; doi:10.1021/jacs.5c10211)
Supplement: Supplementary file 1 [file ja5c10211_si_001.pdf]

Stimuli-responsive luminophore drives mechanism switch for highly efficient electrochemiluminescence immunosensing

Alessandro Fracassa,‡ Giulia Ferrari,‡† Maria Vittoria Balli, Isabella Rimoldi, Giorgio Facchetti, Lorenzo Arnal, Alessia Marconi, Matteo Calvaresi, Luca Prodi, Luisa De Cola,* Giovanni Valenti*

Electronic Supporting Materials

Contents

Chemicals 4

Synthesis and Characterization 5

Synthesis of 4-(4'-methyl-[2,2']bipyridinyl-4-yl)butyric acid succinimidyl ester[1] 5

Synthesis of Tert-butyl (2-((2-aminoethyl)disulfanyl)ethyl)carbamate[2] 5

Synthesis of Tert-butyl(2-((2-(4-(4'-methyl-[2,2'-bipyridin]-4-yl)butanamido)ethyl)disulfanyl) ethyl)carbamate[3] 5

Synthesis of 1-(4'-methyl-[2,2'-bipyridin]-4-yl)-4,13,18,27-tetraoxo-5,12,19,26-tetraazadotriacontan-32-oic acid-methane 6

Synthesis of 1-(4'-methyl-[2,2'-bipyridin]-4-yl)-4,13,18,27-tetraoxo-8,9-dithia-5,12,19,26-tetraazadotriacontan-32-oic acid-methane 6

Synthesis of $Ru(bpy)_2$-(4'-metihyl-(4-tert-butyl-(2-((2-butyramidoethyl)disulfanyl)ethyl)carbamate))bpy ($[Ru(bpy)_2(bpy\text{-}C_4\text{-}cys\text{-}NHBoc)(PF_6)_2]$) 7

Synthesis of $[Ru(bpy)_2(bpy\text{-}C_4\text{-}cys\text{-}NH_2)(PF_6)_2]$, [Ru(S-S)] 7

Synthesis of $[Ru(bpy)_2(bpy\text{-}C_{27}\text{-}COOH)(PF_6)_2]$, $[Ru(C_{27})]$ 8

Synthesis of $[Ru(bpy)_2(bpy\text{-}C_4\text{-}cys\text{-}C_{17}\text{-}COOH)(PF_6)_2]$, $[Ru(S\text{-}S)(C_{21})]$ 8

Beads preparation 9

Covalent functionalization of COOH-coated Beads: $Ru(C_4)$@Bead and Ru(S-S)@Bead 9

Covalent functionalization of biotinylated antibodies $Ru(S\text{-}S)(C_{21})$@Ab and $Ru(C_{27})$@Ab 9

Formation of the immunoassay on magnetic beads $Ru(S\text{-}S)(C_{21})$@Ab-S1-Beads and $Ru(C_{27})$@Ab-S1-Beads 10

Release experiment 10

Electrochemiluminescence 12

ECL microscopy 12

Data Elaboration: Computation of ECL profiles 13

Data elaboration: Integration of the ECL signal 13

COMSOL simulations 14

ECL decay measurements 20

Diffusion of Ru(II) labels in solution 21

ECL imaging at different TPrA concentrations on a GC electrode 22

ECL imaging at different TPrA concentrations on a Pt electrode 24

ECL-TPrA concentration plot on Pt 25

Ru(S-S)@Bead ECL at different TPrA concentrations on GC 26

ECL profiles 26

ECL-TPrA concentration plot on GC 27

Collective beads ECL 30
Literature overview for SARS-CoV-2 detection 31
References..32

Chemicals

Reagents and solvents for synthesis were purchased from Sigma-Aldrich or Zentek and used without further purification. The NMR spectroscopic experiments were carried out either on Varian MERCURY 400 MHz or Bruker Avance I 400 MHz spectrometers (400 and 75 MHz for ^{1}H, ^{13}C and ^{19}F, respectively). All the spectra were recorded at 298 K using TMS as internal standard. HR-MS analyses were performed by using a QTof Synapt G2 Si spectrometer with an electrospray ionization source (Palmer, MA, USA). The MS spectra were obtained by direct infusion of a sample solution of 2 µg/mL in MeOH under ionization, ESI positive.

Bis(2,2'-bipyridine)-[4-(4'-methyl-2,2'-bipyridin-4-yl)butan-1-aminium ruthenium bis(chloride) ($[Ru(bpy)_2(bpy\text{-}C_4\text{-}NH_2)Cl_2]$) was purchased from Cyanagen (Italy) while $[Ru(bpy)_2(bpy\text{-}cys\text{-}NH_2)]^{2+}$ was synthesized. Tris(2,2’-bipyridyl)dichlororuthenium(II) hexahydrate ($[Ru(bpy)_3]Cl_2{\cdot}6H_2O$, MW = 748.62 g mol^{-1}), Tri-*n*-propylamine (TPrA, MW = 143.27 g mol^{-1}, ≥98%), sodium phosphate monobasic dihydrate ($NaH_2PO_4{\cdot}2H_2O$, MW = 156.01 g mol^{-1}, ≥99%), sodium phosphate dibasic (Na_2HPO_4, MW = 141.96 g mol^{-1}, ≥99.5%), phosphoric acid (H_3PO_4, MW = 98.00 g mol^{-1}, ≥85%), sodium 2-(4-morpholinyl)ethanesulfonate (MES, $C_6H_{12}NNaO_4S$, MW = 217.22 g mol^{-1}, ≥99%), sulfuric acid (H_2SO_4, MW = 98.08 g mol^{-1}, 95-98%), N-(3-Dimethylaminopropyl)-N’-ethylcarbodiimide hydrochloride (EDC, $C_8H_{17}N_3{\cdot}xHCl$, MW = 155.24 g mol^{-1}, for synthesis), N-hydroxysulfosuccinimide sodium salt (Sulfo NHS, $C_4H_4NNaO_6S$, MW = 217.13 g mol^{-1}, ≥98%), and dimethyl sulfoxide (DMSO, $(CH_3)_2SO$, MW = 78.13 g mol^{-1}, ≥99.7%) were purchased from Sigma-Aldrich. Carboxy coated 2.8 µm polystyrene beads (Dynabeads™ M-270 Carboxylic Acid) were purchased from Thermo Fisher Scientific Inc.

The immunoassay, targeting inactivated SARS-CoV-2 virus formulated in viral transport medium (AMPLIRUN® TOTAL SARS-CoV-2 CONTROL) purchased from Vircell Microbiologists (Spain), was assembled on Human Anti-SARS-CoV-2 Spike RBD Antibody-coupled 2 µm magnetic beads from ACROBiosystems (Neward, DE, USA). The detection antibody (SARS-CoV-2/2018-nCoV Spike antibody, Rabbit PAb) was purchased from Sino Biological Europe (Germany).

Carbon screen printed electrodes (CSPE, DRP-C11L) were purchased from Metrohm Dropsens (Netherlands). Phosphate buffered saline (PBS, tablet), Bovine serum albumin (BSA, ≥98%), and Tween 20 (d = 1.095 g/mL) were purchased from Sigma-Aldrich.

Synthesis and Characterization

Synthesis of 4-(4'-methyl-[2,2']bipyridinyl-4-yl)butyric acid succinimidyl ester[1]

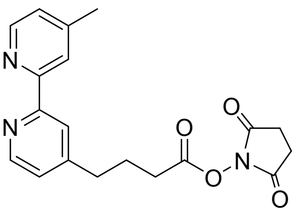

270 mg of DCC (2.34 mmol) and 140 mg H-hydroxysuccinimide (0.98 mmol) were added to 300 mg (1.17 mmol) of 4-(4'-methyl-[2,2']bipyridinyl-4-yl)-butyric acid in CH_2Cl_2 and stirred at room temperature for 2 hours. After 2 hours, the reaction mixture was filtered and concentrated in vacuo and a clear mixture of powder and oil was obtained. After Combi column chromatography (SiO_2, gradient from 100% Hex to 100% EtOAc) the final product was obtained as a clear oil (270 mg, 0.61 mmol, 76%) ^{1}H NMR (300 MHz, CD_3OD): 8.43 (dd, 2H), 8.12 (d, 2H), 7.03 (d, 2H), 2.70 (m, 6H), 2.54 (t, 2H), 2.30 (s, 3H), 2.04 (t, 2H) ppm. ESI-MS: m/z calculated for $C_{19}H_{19}N_3O_4$ 353.38; found 354.47 $[M + H]^+$.

Synthesis of Tert-butyl (2-((2-aminoethyl)disulfanyl)ethyl)carbamate[2]

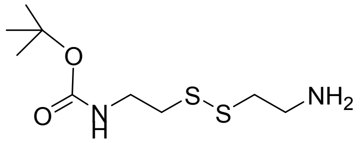

Cystamine dihydrochloride (4 g, 17.78 mmol) was dissolved in methanol (200 mL) and cooled to 0°C. Triethylamine (7.45 mL, 53.33 mmol) was added and stirred for 30 min. Boc-anhydride (4.05 mL, 17.78 mmol) was added dropwise for over 10 min and allowed to stir for 2 h at 0 degrees. The solution was concentrated with a rotary evaporator to get a white precipitate (if it looks like an oil, it's useful to add 60 mL of diethyl ether to let the compound precipitate). Then the white precipitate was filtered and washed three times with 30 mL of diethyl ether. 20 mL of 1 M NaOH solution were added to the product and extracted 2X with CH_2Cl_2 (50 mL). Both organic layers were combined and washed with 2X with water and dried over anhydrous Na_2SO_4 and concentrated in vacuo to yield a yellow oil. (2.4 g, 9.5 mmol, yield 53%). ^{1}H NMR (300 MHz, CD_3OD) δ 3.39 (t, 2H), 3.01 (t, 2H), 2.83 (dd, 4H), 1.48 (s, 9H) ppm. ^{13}C NMR (75 MHz, CD_3OD) δ 158.25, 80.11, 41.46, 40.98, 40.71, 39.07, 28.78 ppm. ESI-MS: m/z $[M + H]^+$ calculated for $C_9H_{20}N_2O_2S_2$ 252.39; found, 253.13.

Synthesis of Tert-butyl(2-((2-(4-(4'-methyl-[2,2'-bipyridin]-4-yl)butanamido)ethyl)disulfanyl) ethyl)carbamate[3]

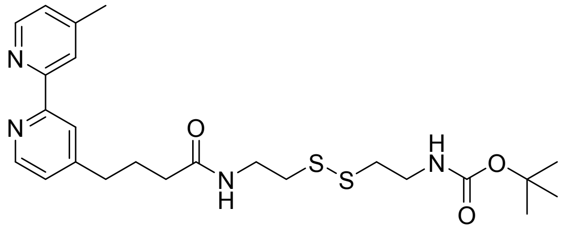

90 mg (0.25 mmol) of 4-(4'-methyl-[2,2']bipyridinyl-4-yl)butyric acid succinimidyl was dissolved in 3 mL DMF, and added to a solution of 100 mg (excess) of Boc-cystamine (0.39 mmol) in 1 mL DMF. After two hours, 0.5 mL of DIPEA was added to ensure deprotonation. The reaction mixture was stirred for 16 hours at room temperature. After 16 hours, the reaction mixture was concentrated in vacuo, taken up in CH_2Cl_2, extracted 2X with a saturated sodium bicarbonate solution, dried over $NaSO_4$, filtered, and re-concentrated in vacuo.

The crude product was washed with saturated NH_4Cl solution to eliminate the excess of cystamine. The light-yellow oil was obtained as the pure product (35 mg, 0.071 mmol, 32%).

^{1}H NMR (300 MHz, CD_2Cl_2) δ 8.48 (dd, 2H), 8.26 (d, 2H), 7.15 (d, 2H), 3.52 (dd, J = 6.0 Hz, 2H), 3.40 (dd, J = 6.5 Hz, 2H), 2.88 – 2.68 (m, 6H), 2.44 (s, 3H), 2.23 (t, J = 7.4 Hz, 2H), 2.01 (t, 2H), 1.40 (s, 9H) ppm. ^{13}C NMR (75 MHz, CD_2Cl_2) δ 172.48, 155.87, 151.66, 149.00, 148.12, 124.62, 123.93, 121.76, 121.11, 79.21, 42.63, 40.64, 38.13, 35.38, 34.63, 28.03, 26.12, 20.96 ppm. MS (ESI) m/z: calculated for $C_{24}H_{34}N_4O_3S_2$ 490.21; found 491.21 $[M +H]^+$.

Synthesis of 1-(4'-methyl-[2,2'-bipyridin]-4-yl)-4,13,18,27-tetraoxo-5,12,19,26-tetraazadotriacontan-32-oic acid-methane

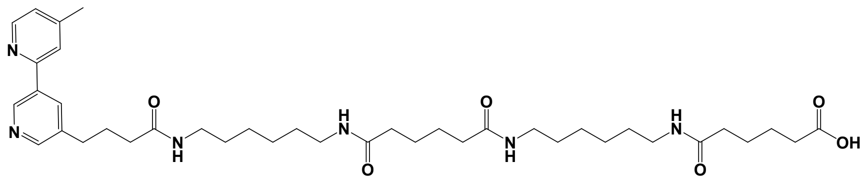

Compound 1-(4'-methyl-[2,2'-bipyridin]-4-yl)-4,13,18,27-tetraoxo-5,12,19,26-tetraazadotriacontan-32-oic acid-methane was prepared by using a cascade reaction starting from 4-(4'-methyl-[2,2']bipyridinyl-4-yl)-butyric acid. 181.41 mg (0.838 mmol) of tert-butyl (6-aminohexyl)carbamate was added to a solution of 181.27 mg of DCC (0.878 mmol), 101.11 mg H-hydroxysuccinimide (0.878 mmol) and 204.7 mg (0.799 mmol) of 4-(4'-methyl-[2,2']bipyridinyl-4-yl)-butyric acid in CH_2Cl_2 and overnight stirred at room temperature. Then, the reaction mixture was filtered and concentrated in vacuo (aprox. 5 mL). That solution was added to a solution of 15 ml of trifluoroacetic acid and 10 mL DCM and let react overnight. Then, the solvent was evaporated under vacuo and 10 mL DCM with 2 mL of triethylamine were added and let react for 30 min, later added it to a solution of 6-((6-((tert-butoxycarbonyl)amino)hexyl)amino)-6-oxohexanoic acid (262.1 mg, 0.761 mmol) in 20 mL of DCM in the presence of 172.7 mg of DCC (0.837 mmol) and 99.33 mg N-hydroxysuccinimide (NHS, 0.837 mmol) and let react overnight. Then, the suspension was filtered thought celite and the solution was washed with a Na_2CO_3 saturated solution and a KH_2PO_4 1 M solution. The organic fraction was dried over anhydrous Na_2SO_4. Subsequently, 20 ml of trifluoroacetic acid and let react overnight. Then, the solvent was evaporated under vacuo to give the product. Immediately after, 2 mL of triethylamine was added and let react for 30 min, next 103.3 mg of adipic anhydride (0.806 mmol). After 18h of reaction the solvent was concentrated under vacuo and a mixture of Et_2O/ Hex (1/1) was added to precipitate the product 1-(4'-methyl-[2,2'-bipyridin]-4-yl)-4,13,18,27-tetraoxo-5,12,19,26-tetraazadotriacontan-32-oic acid-methane (350.4 mg, 0.470 mmol, global yield 59%).

^{1}H NMR (300 MHz, CD_3OD) δ 8.50 (m, 2H), 8.11 (m, 2H), 7.31 (m, 2H), 3.49-3.40 (m, 6H) , 3.05-2.98 (m, 4H), 2.92-2.87 (m, 4H), 2.47 (s, 3H), 2.25-2.22 (m, 2H), 2.19-2.15 (m, 10H), 1.88-1.69 (m, 4H), 1.51-1.46 (m, 8H), 1.39-1.29 (m, 8H) ppm. ^{13}C NMR (75 MHz, CD_3OD) δ 188.40, 174.33, 174.30, 173.88, 173.07, 158.44, 157.16, 152.64, 149.13, 148.75, 148.55, 124.74, 124.09, 122.29, 121.66, 39.82, 39.62, 39.33, 38.93, 38.85, 38.80, 35.38, 34.85, 34.26, 33.34, 32.49, 30.99, 29.46, 28.91, 27.42, 27.38, 27.36, 26.22, 26.16, 25.83, 25.63, 25.24, 24.72, 24.64 ppm. MS (ESI) m/z: calculated for $C_{39}H_{60}N_6O_6$ 708.46; found 707.21 $[M-H]^-$.

Synthesis of 1-(4'-methyl-[2,2'-bipyridin]-4-yl)-4,13,18,27-tetraoxo-8,9-dithia-5,12,19,26-tetraazadotriacontan-32-oic acid-methane

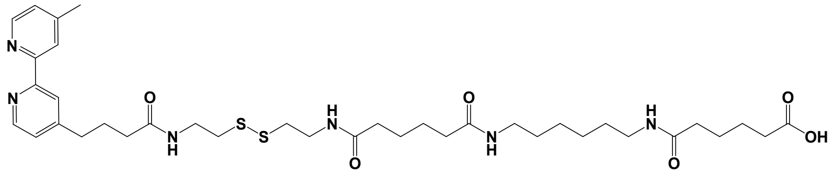

Compound 1-(4'-methyl-[2,2'-bipyridin]-4-yl)-4,13,18,27-tetraoxo-8,9-dithia-5,12,19,26-tetraazadotriacontan-32-oic acid-methane was prepared by using a cascade reaction starting from Tert-butyl(2-((2-(4-(4'-methyl-[2,2'-bipyridin]-4-yl)butanamido)ethyl)disulfanyl) ethyl)carbamate. 304.2 mg of Tert-butyl(2-((2-(4-(4'-methyl-[2,2'-bipyridin]-4-yl)butanamido)ethyl)disulfanyl) ethyl) carbamate (0.778 mmol) was added to a solution of 20 ml of trifluoroacetic acid and 20 mL DCM and let reactant overnight. Then, the solvent was evaporated under vacuo and 10 mL DM with 2 mL of triethylamine were added and let react for 30 min and later added it to a solution of 6-((6-((tert-butoxycarbonyl)amino)hexyl)amino)-6-oxohexanoic acid (255.5 mg, 0.742 mmol) in 20 mL of DCM in the presence of 168.4 mg of DCC (0.815 mmol) and 93.91 mg N-hydroxysuccinimide (NHS, 0.816 mmol) and let react overnight. Then, the suspension was filtered thought celite and the solution was washed with a Na_2CO_3 saturated solution and a KH_2PO_4 1 M solution. The organic fraction was dried over anhydrous Na_2SO_4. Subsequently, 20 ml of trifluoroacetic acid and let react overnight. Then, the solvent was evaporated under vacuo to give the product. Immediately after, 2 mL of triethylamine was added and let react for 30

min, next 100.8 mg of adipic anhydride (0.778 mmol). After 18h of reaction the solvent was concentrated under vacuo and a mixture of Et_2O/ Hex (1/1) was added to precipitate the product 1-(4'-methyl-[2,2'-bipyridin]-4-yl)-4,13,18,27-tetraoxo-8,9-dithia-5,12,19,26-tetraazadotriacontan-32-oic acid-methane (325.6 mg, 0.437 mmol, global yield 56%).
^{1}H NMR (300 MHz, CD_3OD) δ 8.53-8.48 (m, 2H), 8.13-8.11 (m, 2H), 7.30-7.26 (m, 2H), 5.49, 5.19-5.17 (br, 4H), 3.48-3.44 (m, 6H), 2.84-2.73 (m, 8H), 2.44 (s, 3H), 2.32-2.26 (m, 4H), 2.14-2.13 (m, 6H), 1.65-1.59 (m, 10H), 1.37-1.32 (m, 8H). ^{13}C NMR (75 MHz, CD_3OD) δ 189.6, 177.4, 177.3, 177.2, 175.7, 175.3, 171.6, 156.9, 156.7, 153.9, 150.4, 150.1, 149.8, 126.1, 125.5, 123.5, 122.9, 120.1, 116.2, 54.9, 53.5, 51.9, 39.5, 38.4, 36.6, 36.1, 35.5, 35.0, 34.9, 30.8, 27.4, 26.4, 26.3, 25.7, 21.29 ppm. MS (ESI) m/z: calculated for $C_{37}H_{56}N_6O_6S_2$ 744.37; found 744.03 [M-H]$^-$.

Synthesis of Ru(bpy)$_2$-(4'-metihyl-(4-tert-butyl-(2-((2-butyramidoethyl)disulfanyl)ethyl)carbamate))bpy ([Ru(bpy)$_2$(bpy-C$_4$-cys-NHBoc)(PF$_6$)$_2$])

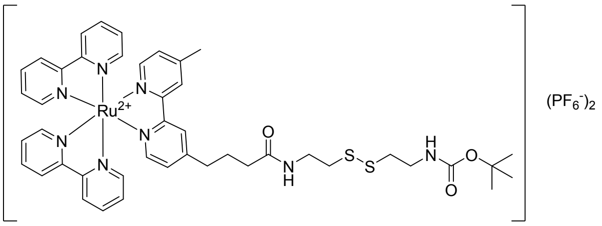

In a single neck round bottom flask *cis*-bis-(2,2'-bipyridine)-dichlororuthenium (II) (35 mg, 0.071 mmol) and $AgPF_6$ (36 mg, 0.142 mmol) were dissolved in acetone (15 mL). The reaction mixture was allowed to stir at room temperature for 8 h. To this mixture tert-butyl (2-((2-(4-(4'-methyl-[2,2'-bipyridin]-4-yl)butanamido)ethyl)disulfanyl) ethyl)carbamate (35 mg, 0.071 mmol) was added and refluxed for 24 h). The solvent was filtered twice to remove all the precipitated AgCl and dried under high vacuum. Et_2O was added dropwise, and the orange precipitate was collected by vacuum filtration and then dried under high vacuum. The product was obtained as a red-brown powder (31 mg, 0.034 mmol, yield 48%).
^{1}H NMR (300 MHz, CD_3CN) δ 8.57 – 8.43 (d, 4H), 8.37 (d, J = 11.0 Hz, 2H), 8.15 – 7.99 (m, 4H), 7.73 (d, J = 3.3 Hz, 4H), 7.67 – 7.50 (m, 2H), 7.50 – 7.33 (m, 4H), 7.33 – 7.19 (m, 2H), 3.39 (q, J = 6.5 Hz, 2H), 3.29 (q, J = 6.3 Hz, 2H), 2.88 – 2.69 (m, 6H), 2.53 (s, 3H), 2.22 (t, J = 7.3 Hz, 4H), 1.38 (s, 9H) ppm. ^{13}C NMR (101 MHz, CD_3CN) δ 172.16, 157.08, 156.51, 154.07, 153.33, 152.15, 151.69, 150.76, 150.47, 138.35, 137.97, 137.62, 136.85, 128.31, 127.52, 126.73, 125.03, 124.20, 123.56, 122.88, 117.32, 78.54, 39.41, 37.94, 34.80, 34.14, 27.63, 25.68, 20.28 ppm. MS (ESI) m/z: calculated for $C_{44}H_{50}N_8O_3RuS_2^{2+}$ 904.13/2; found 452.35 [M/2]$^+$. Elemental analysis: C% calculated 44.3, found 43.9; H% calculated 4.2, found 4.0; N% calculated 9.4, found 9.3.

Synthesis of [Ru(bpy)$_2$(bpy-C$_4$-cys-NH$_2$)(PF$_6$)$_2$], [Ru(S-S)]

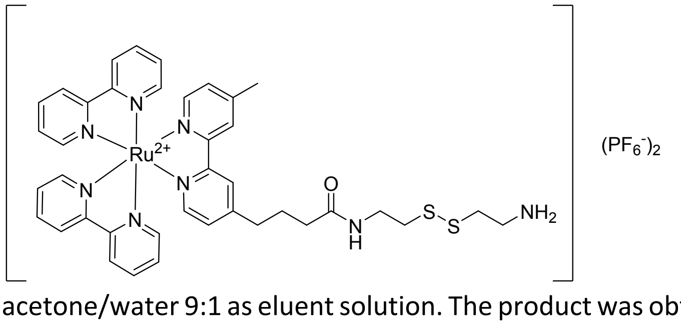

Complex [Ru(bpy)$_2$(bpy-cys-NHBoc)(PF$_6$)$_2$] (100 mg, 0.11 mmol) was dissolved in 12 ml CH_3CN/CH_2Cl_2 1:1. The same quantity of TFA was then added to the solution (12 ml, 184.30 mmol) and the reaction stirred overnight at room temperature in air. Solvents are rotary evaporated, and the purification was done with neutral alumina chromatography column with acetone/water 9:1 as eluent solution. The product was obtained as an orange-red powder (50 mg, 0.05 mmol, yield: 45%).
^{1}H NMR (300 MHz, CD_3CN) δ 8.55 (dd, J = 8.7, 2.9 Hz, 6H), 8.10 – 7.99 (m, 4H), 7.74 (q, J = 7.0 Hz, 4H), 7.56 – 7.50 (m, 2H), 7.48 – 7.34 (m, 4H), 7.29 – 7.19 (m, 2H), 3.36 – 3.20 (m, 2H), 2.96 – 2.84 (m, 2H), 2.84 – 2.62 (m, 6H), 2.52 (d, J = 3.6 Hz, 3H), 2.25 (t, J = 7.6 Hz, 3H) ppm. ^{13}C NMR (101 MHz, CD_3CN) δ 181.04, 157.67, 155.11, 152.27, 151.48, 151.33, 138.20, 128.85, 128.25, 128.10, 125.64, 124.91, 124.80, 117.90, 45.38, 39.24, 35.52, 34.85, 33.82, 32.18, 29.51, 26.35, 20.83 ppm. ^{19}F NMR (282 MHz, CD_3CN) δ -72.80 (d, *J* = 705 Hz, 2PF$_6^-$) ppm. FT-IR ν(cm^{-1}) = 3423, 2963, 2188, 2078, 1681, 1422, 1204, 1132, 1091, 838. MS (ESI) m/z: calculated for $C_{39}H_{41}N_8ORuS_2^{\ 2+}$ 804.01/2; found 402.30 [M/2]$^+$. Elemental analysis: C% calculated 39.2, found 38.8; H% calculated 3.4, found 3.4; N% calculated 9.4, found 9.3.

Synthesis of $[Ru(bpy)_2(bpy\text{-}C_{27}\text{-}COOH)(PF_6)_2]$, $[Ru(C_{27})]$

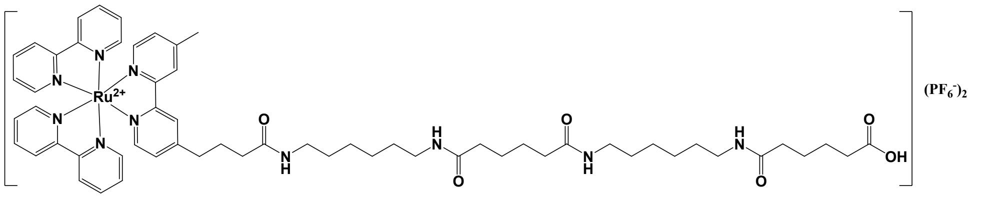

In a single neck round bottom flask *cis*-bis-(2,2'-bipyridine)-dichlororuthenium (II) (68.32 mg, 0.141 mmol) and $AgPF_6$ (71.33 mg, 0.282 mmol) were dissolved in acetone (15 mL). The reaction mixture was allowed to stir at room temperature for 8 h. To this mixture 1-(4'-methyl-[2,2'-bipyridin]-4-yl)-4,13,18,27-tetraoxo-5,12,19,26-tetraazadotriacontan-32-oic acid-methane (100 mg, 0.141 mmol) was added and let react overnight. The solvent was filtered twice to remove all the precipitated AgCl and dried under high vacuum. The purification was done with neutral alumina chromatography column with acetone as eluent solution and then water to elute the product that remains in the column. The product was obtained as a red-brown powder (95.3 mg, 0.067 mmol, yield 48%).

^1H NMR (600 MHz, CD_3CN) δ 9.70 (br, 1H), 8.55-8.51 (m, 6H), 8.40 (d, *J* = 11.0 Hz, 2H), 8.09-8.05 (m, 4H), 7.77-7.73 (m, 4H), 7.58-7.55 (m, 2H), 7.44-7.39 (m, 2H), 7.29-7.25 (m, 2H), 3.45-3.39 (m, 2H), 3.15- 3.11 (m, 8H), 2.60 (s, 3H), 2.56-2.54 (m, 2H), 2.31-2.28 (m, 8H), 1.61-1.58 (m, 10H), 1.31-1.25 (m, 16H) ppm. ^{13}C NMR (151 MHz, CD_3CN) δ 175.42, 159.33, 158.54, 158.43, 158.35, 158.00, 157.86, 155.38, 154.67, 153.50, 153.05, 152.91, 152.29, 152.11, 151.81, 139.70, 139.32, 139.16, 138.97, 129.65, 128.94, 128.88, 128.08, 126.39, 125.63, 125.56, 125.25, 124.92, 47.58, 39.30, 38.92, 37.24, 36.19, 35.49, 34.79, 34.35, 30.72, 29.08, 27.08, 26.89, 26.79, 26.52, 26.13, 25.43, 23.76, 21.62, 14.76 ppm. ^{19}F NMR (565 MHz, CD_3CN) δ -72.85 (d, *J* = 706 Hz, $2PF_6^-$) ppm. MS (ESI) m/z: calculated for $C_{59}H_{76}N_{10}O_6Ru^{2+}$ 1122.50/2; found 1121.49 $[M]^+$. found 561.25 $[M/2]^+$. Elemental analysis: C% calculated 63.1, found 63.7; H% calculated 6.8, found 6.9; N% calculated 12.5, found 12.2.

Synthesis of $[Ru(bpy)_2(bpy\text{-}C_4\text{-}cys\text{-}C_{17}\text{-}COOH)(PF_6)_2]$, $[Ru(S\text{-}S)(C_{21})]$

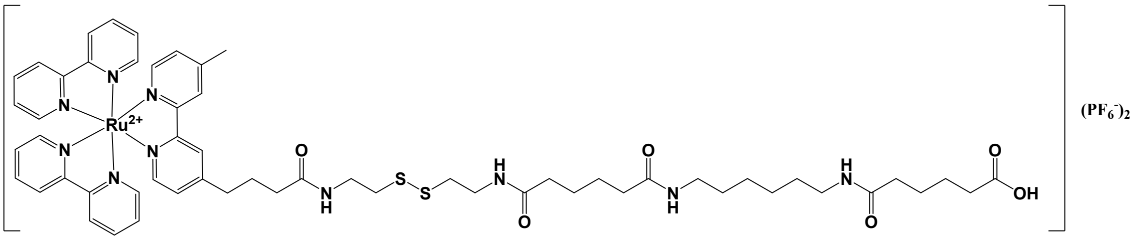

In a single neck round bottom flask *cis*-bis-(2,2'-bipyridine)-dichlororuthenium (II) (65.01 mg, 0.134 mmol) and $AgPF_6$ (67.87 mg, 0.268 mmol) were dissolved in acetone (15 mL). The reaction mixture was allowed to stir at room temperature for 8 h. To this mixture 1-(4'-methyl-[2,2'-bipyridin]-4-yl)-4,13,18,27-tetraoxo-8,9-dithia-5,12,19,26-tetraazadotriacontan-32-oic acid-methane (100 mg, 0.134 mmol) was added and let react overnight. The solvent was filtered twice to remove all the precipitated AgCl and dried under high vacuum. The purification was done with neutral alumina chromatography column with acetone as eluent solution and then water to eluate the product that remains in the column. The product was obtained as a red-brown powder (101.6 mg, 0.070 mmol, yield 52%).

^1H NMR (600 MHz, CD_3CN) δ 9.35 (br, 1H), 8.57-8.55 (m, 6H), 8.42 (d, J = 12.0 Hz, 2H), 8.29 (t, J = 11.0 Hz, 2H), 8.09-8.05 (m, 2H), 7.97 (t, J = 11.0 Hz, 2H), 7.88 (t, J = 12.0 Hz, 2H), 7.75-7.72 (m, 2H), 7.61 (t, J = 11.0 Hz, 2H), 7.41-7.37 (m, 2H), 3.60-3.56 (m, 6H), 3.14-3.10 (m, 4H), 2.58-2.52 (m, 4H), 2.31 (s, 3H), 2.30-2.28 (m, 2H), 1.99-1.96 (m, 8H), 1.60-1.55 (m, 2H), 1.37-1.28 (m, 16H) ppm. ^{13}C NMR (151 MHz, CD_3CN) δ 175.93, 162.10, 161.88, 161.66, 161.44, 159.34, 158.53, 158.44, 154.68, 153.49, 153.03, 139.70, 139.32, 139.17, 138.97, 128.88, 128.09, 127.18, 125.59, 125.27, 124.94, 121.49, 61.35, 59.44, 58.31, 47.30, 43.08, 41.05, 40.02, 35.0, 34.8, 34.68, 34.12, 33.59, 30.76, 30.47, 27.43, 27.25, 26.70, 26.75, 26.53, 25.89, 25.57, 21.52,

14.93, 14.89, 13.83. ^{19}F NMR (565 MHz, CD_3CN) δ -72.82 (d, *J* = 701 Hz, $2PF_6^-$) ppm. HR-MS (ESI) m/z: calculated for $C_{57}H_{72}N_{10}O_6RuS_2^{2+}$ 1158.41/2; found 1157.40 $[M]^+$, 579.20 $[M/2]^+$. Elemental analysis: C% calculated 59.1, found 59.5; H% calculated 6.3, found 6.2; N% calculated 12.1, found 12.0.

Beads preparation

Covalent functionalization of COOH-coated Beads: Ru(C_4)@Bead and Ru(S-S)@Bead

5 µL of beads stock suspension (30 mg/mL) were washed three times with 200 µL of 25 mM MES buffer (pH 6) using magnetic support. Subsequently, 450 µL of a freshly prepared solution of EDC (12.5 mM) and Sulfo NHS (25 mM) in MES buffer were added to the beads suspension. The mixture was incubated for 30 minutes at room temperature under physical mixing at 1100 rpm by ZX3 Advanced Vortex Mixer. After carboxy groups activation step, the supernatant was retrieved and 187.5 µL of a 1 mM solution of Ru(II) complex (5 Ru(II) complex equivalents per COOH) in a 80:20 mixture of 0.3 M PB (pH 6.8) and DMSO were added to the beads suspension. The mixture was incubated overnight at room temperature under physical mixing at 1100 rpm. After incubation, the beads were washed with 200 µL of PB with <0.1% surfactant for 5 times using magnetic support. Finally, Ru(II)-labeled beads were suspended in PB with <0.1% surfactant to a final concentration of 0.72 mg/mL and stored at 4 °C.

Covalent functionalization of COOH-coated Beads for Release Experiment: 144 µL of beads stock suspension (30 mg/mL) were washed three times with 200 µL of 25 mM MES buffer (pH 6) using magnetic support (PureProteome™ Magnetic Stand, Sigma Aldrich). Subsequently, 4 mL of a freshly prepared solution of EDC (12.5 mM) and Sulfo NHS (25 mM) in MES buffer were added to the beads suspension. The mixture was incubated for 30 minutes at room temperature under physical mixing at 1100 rpm by ZX3 Advanced Vortex Mixer (VELP Scientifica Srl, Italy). After carboxy groups activation step, the supernatant was retrieved and 5.4 mL of a 1 mM solution of Ru(II) complex (5 Ru(II) complex equivalents per COOH) in a 80:20 mixture of 0.3 M PB (pH 6.8) and DMSO were added to the beads suspension. The mixture was incubated overnight at room temperature under physical mixing at 1100 rpm. After incubation, the beads were washed with 200 µL of PB with <0.1% surfactant for 5 times using magnetic support. Finally, Ru(II)-labeled beads were suspended in PB with <0.1% surfactant to a final volume of 500 µL (8.64 mg/mL) and stored at 4 °C.

Covalent functionalization of biotinylated antibodies Ru(S-S)(C_{21})@Ab and Ru(C_{27})@Ab

To prevent antibody oligomerization, which could result from the activation of the aspartate/glutamate of the antibody for the conjugation to the amine terminal $[Ru(bpy)_2(bpy\text{-}cys\text{-}NHBoc)]^{2+}$, terminal COOH derivatives of both conventional and cleavable Ru(II) complexes were synthesized named [Ru(C_{27})] and [Ru(S-S)(C_{21})].
Terminal COOH Ru(II) complexes were activated by incubating a 17 mM solution with EDC (17 mM) and Sulfo NHS (25 mM) in DMSO for three hours at 37 °C with continuous mixing. Subsequently, 85 equivalents of activated Ru(II) were added to an antibody solution in 1x PBS to a final antibody concentration of 0.1 mg/mL. The mixture was incubated for 1 hour at 37 °C under physical mixing at 700 rpm. Finally, Ru(II)-conjugated antibodies were purified via dialysis in 1x PBS.

Formation of the immunoassay on magnetic beads Ru(S-S)(C_{21})@Ab-S1-Beads and Ru(C_{27})@Ab-S1-Beads

The ECL bead-based assay involves three sequential procedures: I) a preliminary blocking-coating procedure of the Human Anti-SARS-CoV-2 Spike RBD Antibody-coupled magnetic beads; II) an immunoassay procedure (in which the classical sequential incubations for the immuno-recognition events are merged in a single incubation of 10 min); III) detection of the ECL signal using screen printed electrodes (SPE, DRP-C11L) and a photomultiplier tube (PMT).

I) Blocking-coating procedure: A total of 6 µL of bead stock suspension (0.72 mg/mL) was transferred into a 1.5 mL Eppendorf tube and washed twice with 180 µL of washing buffer (1× PBS, pH 7.4, containing 0.05% Tween 20). The beads were then blocked by incubating them in 200 µL of blocking buffer (washing buffer supplemented with 1.5% BSA) for 30 minutes at room temperature (RT). After incubation, the supernatant was discarded, and the beads were washed twice with 180 µL of washing buffer. For each washing step, the tube was placed on a magnetic rack for 30 seconds to allow bead separation, followed by removal of the supernatant.

II) Immunoassay procedure: Thirty bead samples were prepared at decreasing virion concentrations—fifteen samples for each type of Ru(II)-derivatized antibody, with three replicates per concentration. To each sample of pre-washed and blocked beads, varying volumes of whole virus solution in swab formulation (250 cps/µL) were added: 2 µL, 1 µL, 0.5 µL, 0.3 µL, and 0 µL. This was followed by the addition of 3 µL of labeled detection antibody (30.75 nM). The total volume was adjusted to 250 µL using washing buffer. The samples were incubated for 10 minutes at RT. Subsequently, the beads were washed twice with 180 µL of washing buffer and finally resuspended in 6 µL of washing buffer to maintain the original bead concentration. The samples were stored at 4 °C until use. As before, each washing step included a 30-second magnetic separation followed by removal of the supernatant.

III) ECL measurements: Following the immunoassay, the entire bead suspension—carrying the immunological complex—was drop cast onto a screen-printed working electrode. Beads were magnetically concentrated onto the electrode surface using a magnet placed underneath. The counter electrode was a platinum wire, while a Ag/AgCl was used as the reference electrode. This three-electrode system was placed in a 2x1 cm glass cuvette and housed in a dark box to eliminate interference from ambient light. The cell was carefully placed as close as possible to the PMT (H13543-20, Hamamatsu Photonics K.K., Japan), which was positioned in front of the working electrode. The ECL signal, generated during a two-step chronoamperometry (E_1: open circuit potential for 2 s; E_2: 1.9 V for 11 s), was collected by the PMT and amplified with a Keithley Model 6485 Picoammeter (Keithley Instruments Inc., United States).

Release experiment

The release study, designed to quantify the amount of Ru(II) cleaved from the bead surface, was conducted using Ru(S-S)@Bead deposited on a glassy carbon (GC) electrode (Fig. S1). The setup included a working electrode, an Ag/AgCl (Sat'd KCl) reference electrode, and a Pt wire counter electrode, all housed in a three-electrode electrochemical cell containing 1 mL of 0.3 M phosphate buffer (PB) solution with 180 mM TPrA (pH 6.8).

A single measurement cycle involves a two-step chronoamperometry consisting in an initial step at 0 V followed by a second step at 1.4 V. This potential ensures diffusion-controlled oxidation of TPrA at the GC electrode (Fig. S2). Following this step, the coreactant solution is recovered, the electrode surface is meticulously cleaned, and the cycle is completed. The release experiment consists of ten such cycles. Each cycle utilizes fresh, functionalized beads on the electrode surface, while the same coreactant solution is employed through-out the whole experiment. This approach concentrates the luminophore in solution, facilitating its detection by ICP-MS.

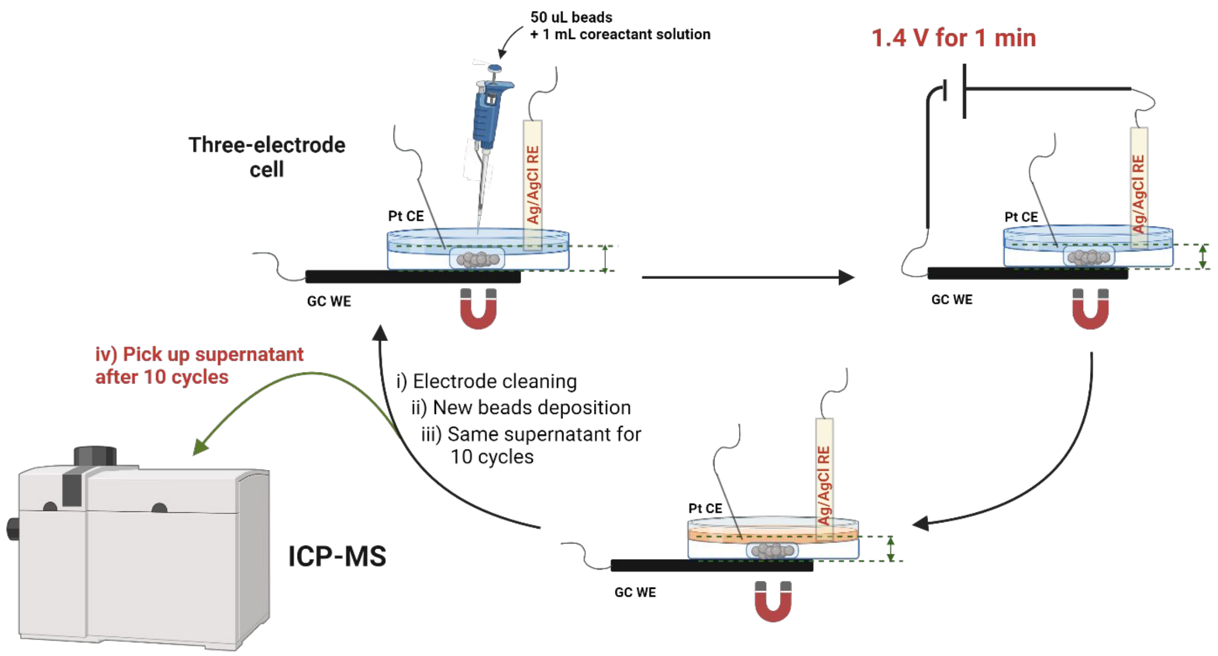

Figure S1. Schematic representation of the whole release experiment towards accurate quantification of Ru released in a coreactant solution after Ru(S-S)@Bead cleavage.

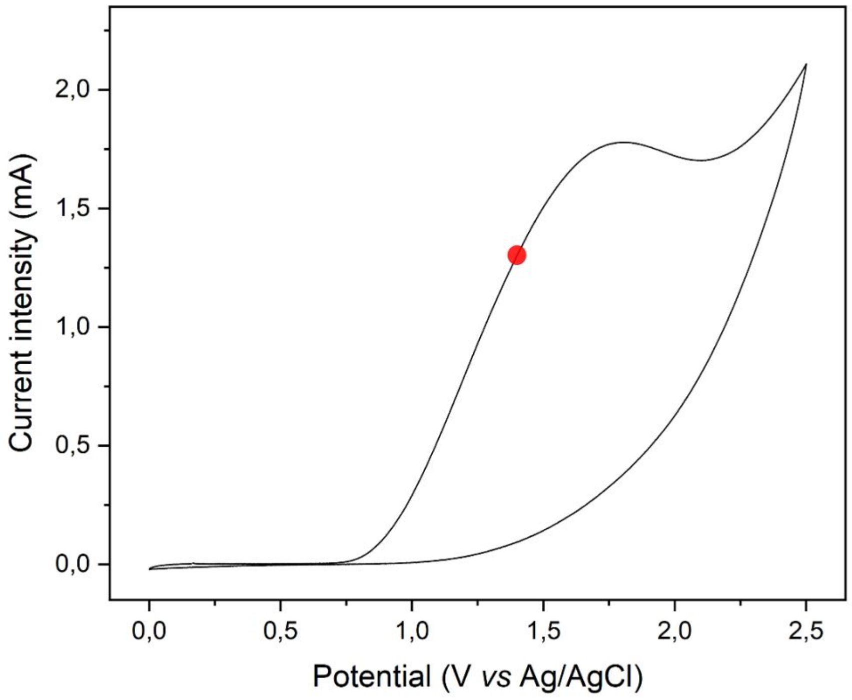

Figure S2. Current-potential curve of a 180 mM TPrA solution at 100 mV·s^{-1} on a GC electrode. The red circle highlights the current intensity at 1.4 V, the potential that was applied during the release experiment.

Electrochemiluminescence

ECL microscopy

In the microscopy setup, the ECL and optical images were captured following the injection of a suspension of Ru(II) covalently functionalized beads in the electrochemical cell where the microspheres were collected on the working electrode surface using a magnet placed underneath. The ECL and optical imaging was performed using solutions of 0.3 M PB with variable TPrA concentrations (pH 6.8), in a PTFE homemade electrochemical cell comprising a GC working (3.5 cm^2), Pt counter, and Ag/AgCl (3 M KCl) reference electrodes. The different solutions were inserted in the electrochemical cell with a pressure-driven flow controller (OB1 Mk3, Elveflow) equipped with a flux sensor (Flow-04D working range from 0 to 1000 µL/min) and exchanged, when necessary, with a 10-way bidirectional valve (MUX distributor). For microscopic imaging, an epifluorescence microscope from Nikon (Chiyoda, Tokyo, Japan) equipped with an ultrasensitive EMCCD camera (EM-CCD 9100−13 from Hamamatsu, Japan) was used with a resolution of 512 × 512 pixel and a size of 16 × 16 μm^2. The microscope was enclosed in a homemade dark box to avoid interferences from external light. It was also equipped with a motorized microscope stage (Corvus, Märzhauser, Wetzlar, Germany) for sample positioning and with water-dipping objective from Nikon (magnification 100×/NA 1.10/WD (mm) 2.5). Additionally, the integrated system included a SP-300 potentiostat (BioLogic Science Instrument, France) triggered with the camera.

ECL images were captured during a double chronoamperometric pulse: 0 V (*vs* Ag/AgCl 3M KCl) for 2 s, followed by 2.5 V (*vs* Ag/AgCl 3M KCl) for varying durations, as detailed in the caption of the respective images. The total integration time of the EM-CCD camera was set to cover the entire span of the measurement.

ECL signal decay were monitored by capturing a frame every 500 ms during a 13 s two-step chronoamperometry: 2 s at 0 V vs Ag/AgCl, followed by 11 s at 2.5 V vs Ag/AgCl.

Unless otherwise stated, gain and sensitivity parameters of the EM-CCD camera were set to 1 and 250, respectively.

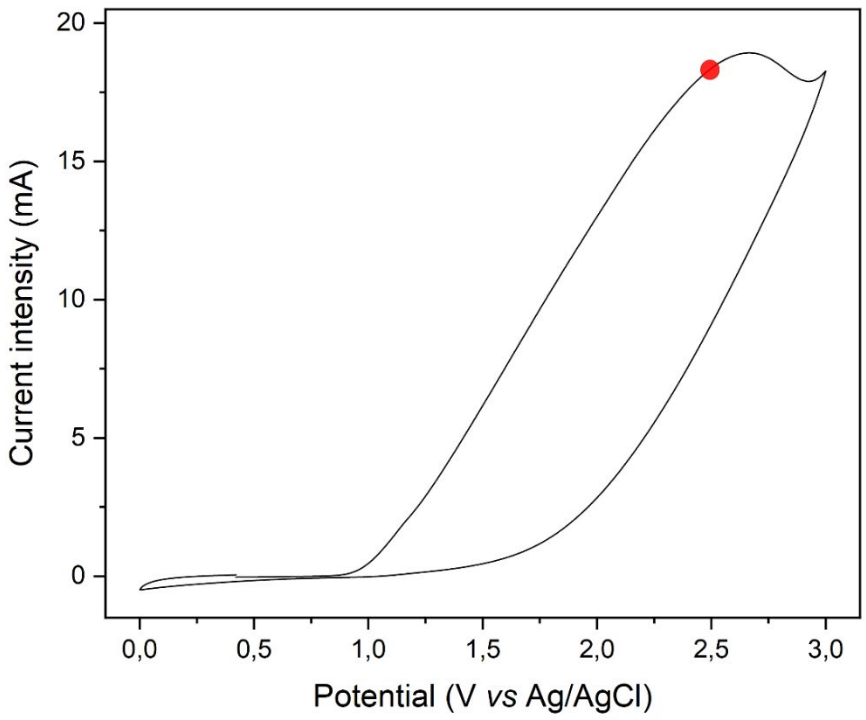

Figure S3. Current-potential curve of a 180 mM TPrA solution at 100 mV·s^{-1} on a GC electrode. The red circle highlights the current intensity at 2.5 V, the potential that was applied during the two-step chronoamperometry to trigger the ECL emission.

Data Elaboration: Computation of ECL profiles

The ECL intensity profiles were extracted using the default Plot profile setting of the ImageJ software from a 90x4 pixel region of interest (ROI, 14.28x0.63 µm) centred on the bead as shown in Fig. 2 in the main text. Thus, the software generates a profile where the signal intensity for each pixel along the x-axis (i.e., distance) is averaged over the signal intensity of 4 adjacent pixels that lie at the same x value.

During data processing, the background noise, arising from the thermal dark current of the EM-CCD camera at its operating temperature (-45 °C), was subtracted from the ECL profiles (Fig. S4). Since the emission of Ru(C_4)@Bead occurs exclusively at the beads surface (i.e., emission from attached $[Ru(bpy)_3]^{2+}$ labels), the dark noise was determined by averaging the signal intensity of the first 10 pixels (1.59 µm), where no bead emission was detected. This average value was used as a baseline and subtracted from each point of the raw ECL profiles. However, this approach is not feasible for Ru(S-S)@Bead as it presents homogeneous ECL emission in solution that overlaps with the dark noise. Nevertheless, under the same experimental conditions, it is reasonable to assume that Ru(S-S)@Bead and Ru(C_4)@Bead share the same average background noise. Thus, the baseline used for background subtraction with Ru(C_4)@Bead, was also applied to the corresponding ECL measurements on Ru(S-S)@Bead.

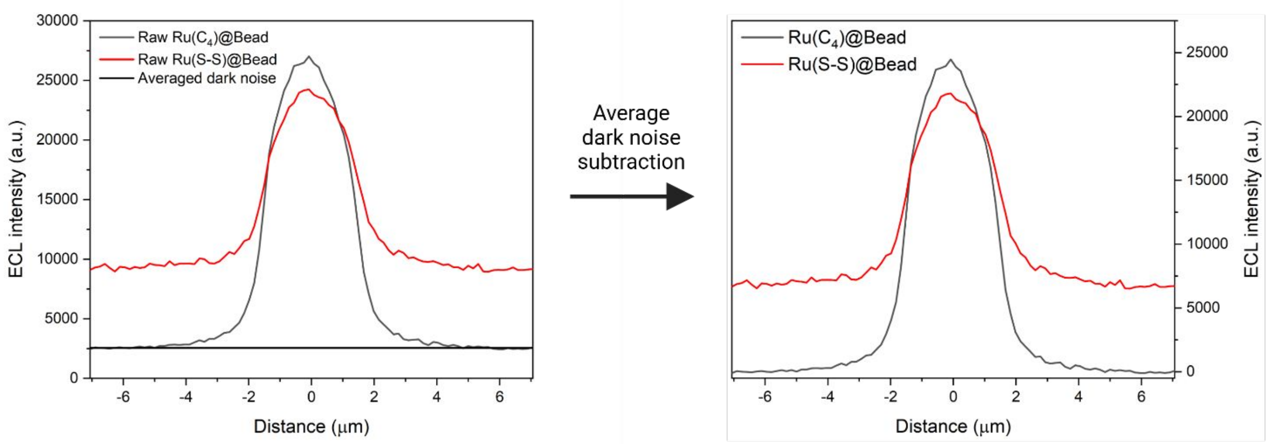

Figure S4. Scheme illustrating the processing of ECL profile data for the background noise subtraction in Ru(C_4)@Bead and Ru(S-S)@Bead.

Data elaboration: Integration of the ECL signal

The ECL intensity, whether from a given frame of a transient measurement or a single integrated image, was calculated over a 50x50 pixels ROI (7.94x7.94 µm) centered on the bead. This area includes not only the bead itself, but also its immediate chemical environment to account for the contribution of homogeneous ECL beyond the bead edges. The raw ECL value was obtained by summing the ECL intensity of all pixels within the ROI (i.e., integration). For Ru(C_4)@Bead, the background noise was determined by integrating the signal over a 7.94x7.94 µm ROI centered on a region where no bead emission is detected. The resulting dark noise value was then subtracted to the raw ECL value. Similarly to the previous case, the background noise for Ru(S-S)@Bead was assumed to be the same as that of its non-cleavable counterpart.

COMSOL simulations

Finite element simulations were carried out on a single bead by the commercial software COMSOL Multiphysics (Version 6.1). In this simulation, a 2D axisymmetric geometry was built to simulate the physical geometry of the substrate used in ECL experiments (Fig. S8a), with a refined mesh at the electrode surface and the bead surface boundaries (Fig. S8b). The bulk boundary is set at a constant concentration of TPrA (180 mM). “Transport of Diluted Species” physical field was employed for studying the time-dependent transport of diluted species involved in ECL reactions. Instead, $[Ru(bpy)_3]^{2+}$ labels attached to the bead surface and reactions at the bead/electrolyte interface are described in the “Surface Reactions” physical field.

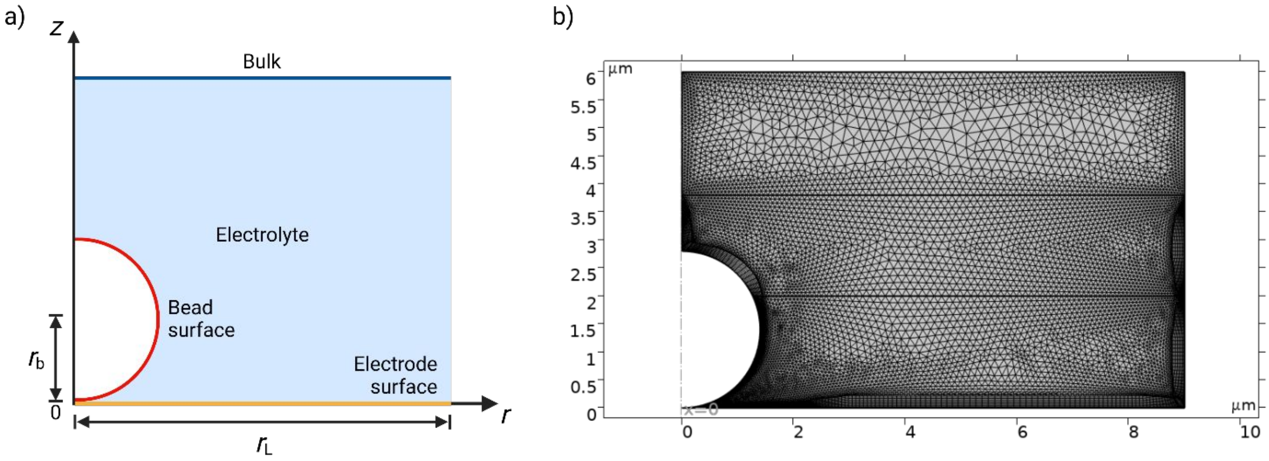

Figure S5. a) 2D axisymmetric model employed in the COMSOL simulation (not to real scale). r and z coordinates represent the parallel and normal directions to the electrode surface, respectively. r_b represents the radius of the bead. r_L represents the length of the electrode. b) Mesh settings for COMSOL simulation with refinements at the electrode and bead boundaries.

The model aims to simulate the limiting case where Ru(II) labels bound to the bead does not participate in any ECL reaction but can only be cleaved by reacting with $TPrA^{\bullet}$. Therefore, on the bead surface, we accounted only for a single redox state of the luminophore, namely $[Ru(bpy)_3]^{2+}{}_{(bound)}$. On the other hand, the reactive species considered in solution include all the redox-active states of the luminophore and the coreactant involved in the generation of the ECL signal. Namely, the simulation encompasses $[Ru(bpy)_3]^{2+}$, and its oxidized and reduced forms $[Ru(bpy)_3]^{3+}$ and $[Ru(bpy)_3]^{+}$, respectively; the neutral TPrA and its protonated form $TPrAH^{+}$, its radical cation $TPrA^{\bullet+}$, and the neutral radical $TPrA^{\bullet}$.
Equations describing all the electrochemical and chemical reaction steps incorporated in the model are provided in Table S1. All parameters used in the simulation are summarized in Table S2.

Table S1. Reactions involved in the simulation

Category	Reaction Equation	
Charge Transfer Reactions	$TPrA \xrightarrow{ko} TPrA^{\bullet +} + e^-$	(1)
	$TPrA^{\bullet} \xrightarrow{ko} Im^+ + e^-$	(2)
	$[Ru(bpy)_3]^{2+} \xrightarrow{koRu} [Ru(bpy)_3]^{3+} + e^-$	(3)
	$[Ru(bpy)_3]^{+} \xrightarrow{koRu} [Ru(bpy)_3]^{2+} + e^-$	(4)
Homogeneous Reactions	$TPrAH^+ \underset{k1}{\overset{k1\_}{\rightleftarrows}} TPrA + H^+$	(5)
	$TPrA^{\bullet +} \underset{k3}{\overset{k3\_}{\rightleftarrows}} TPrA^{\bullet} + H^+$	(6)
	$TPrA^{\bullet} + [Ru(bpy)_3]^{3+} \xrightarrow{kEG3} Im^+ + [Ru(bpy)_3]^{2+*}$	(7)
	$TPrA^{\bullet} + [Ru(bpy)_3]^{2+} \xrightarrow{kEG} Im^+ + [Ru(bpy)_3]^{+}$	(8)
	$TPrA^{\bullet +} + [Ru(bpy)_3]^{+} \xrightarrow{kIG1} TPrA + [Ru(bpy)_3]^{2+*}$	(9)
	$[Ru(bpy)_3]^{3+} + [Ru(bpy)_3]^{+} \xrightarrow{kG1G3} [Ru(bpy)_3]^{2+} + [Ru(bpy)_3]^{2+*}$	(10)
	$TPrA + [Ru(bpy)_3]^{3+} \xrightarrow{kAG3} TPrA^{\bullet +} + [Ru(bpy)_3]^{2+}$	(11)
	$TPrA^{\bullet +} + TPrA^{\bullet} \xrightarrow{kEI} TPrA + Im^+$	(12)
	$[Ru(bpy)_3]^{+} \xrightarrow{kG1} [Ru(bpy)_3]^{2+}$	(13)
Reaction on the bead surface	$[Ru(bpy)_3]^{2+}_{(bound)} + TPrA^{\bullet} \xrightarrow{kSS} [Ru(bpy)_3]^{2+}_{(free)} + Im^+$	(14)

Table S2. A summary of simulation parameters

Name	Value	Description
alpha	0.5	Electron transfer coefficient
F	96485 $[C\cdot mol^{-1}]$	Faraday constant
R	8.314 $[J\cdot mol^{-1}\cdot K^{-1}]$	Gas constant
T	298.15 [K]	Temperature
f_a	38.92 $[V^{-1}]$	F/RT
n	1	n° of electrons exchanged
N_a	$6.02\cdot 10^{23}$ $[mol^{-1}]$	Avogadro number
E_a	1.4 [V]	Applied potential
E^o	0.9 [V]	TPrA standard oxidation potential
$E^o{}_d$	-1.7 [V]	TPrA radical standard oxidation potential
$E^o{}_{Ru}$	1.1 [V]	$[Ru(bpy)_3]^{2+}$ standard oxidation potential
$E^o{}_{Ru\_}$	-1.3 [V]	$[Ru(bpy)_3]^{2+}$ standard reduction potential
D	$5\cdot 10^{-6}[cm^2\cdot s^{-1}]$	Diffusion coefficient for TPrA and its radicals
D_H	$9.3\cdot 10^{-5}[cm^2\cdot s^{-1}]$	Diffusion coefficient for H^+
D_{Ru}	$5.9\cdot 10^{-6}[cm^2\cdot s^{-1}]$	Diffusion coefficient for $[Ru(bpy)_3]^{2+}$
D_{min}	$1\cdot 10^{-50}$ $[cm^2\cdot s^{-1}]$	Diffusion coefficient for hν
k_o	Variable	Rate constant at zero potential for TPrA ET
k_{oRu}	10 $[cm\cdot s^{-1}]$	Rate constant at zero potential for $[Ru(bpy)_3]^{2+}$ ET
$k_{1\_}$	$8\cdot 10^{-3}$ $[s^{-1}]$	Forward rate constant Eq. 5
k_1	$1\cdot 10^{7}$ $[M^{-1}\cdot s^{-1}]$	Backward rate constant Eq. 5
K	k1/k1_	
$k_{3\_}$	$3.5\cdot 10^{3}$ $[s^{-1}]$	Forward rate constant Eq. 6
k_3	$1\cdot 10^{6}$ $[M^{-1}\cdot s^{-1}]$	Backward rate constant Eq. 6
kEG3	$1\cdot 10^{9}$ $[M^{-1}\cdot s^{-1}]$	Rate constant Eq. 7
kEG	$1\cdot 10^{6}$ $[M^{-1}\cdot s^{-1}]$	Rate constant Eq. 8
kIG1	$1\cdot 10^{6}$ $[M^{-1}\cdot s^{-1}]$	Rate constant Eq. 9
kG1G3	$1\cdot 10^{7}$ $[M^{-1}\cdot s^{-1}]$	Rate constant Eq. 10
kAG3	$5\cdot 10^{4}$ $[M^{-1}\cdot s^{-1}]$	Rate constant Eq. 11
kEI	$1\cdot 10^{7}$ $[M^{-1}\cdot s^{-1}]$	Rate constant Eq. 12
kG1	200 $[s^{-1}]$	Rate constant Eq. 13
kSS	Variable	Rate constant Eq. 14
k_{em}	$1\cdot 10^{6}$ $[s^{-1}]$	Photon emission rate
H_0	$1\cdot 10^{-7}$ [M]	Initial $[H^+]$ at pH 7
A_{0t}	$180\cdot 10^{-3}$ [M]	Initial [TPrA]
CC_0	$\mathrm{A0t}\Big/1+\frac{1}{\mathrm{K\cdot H0}}$	Analytical $[TPrAH^+]$
A_0	A0t-CC0	Analytical [TPrA]
C_0	0	Initial concentration = 0
Ru_0	$2.1\cdot 10^{-5}$ $[mol\cdot m^{-2}]$	Initial concentration of $[Ru(bpy)_3]^{2+}$ on the bead
R_b	1.4 [µm]	Bead radius

The rate constants for heterogeneous oxidation at the studied overpotential (E_a), modeled according to Butler-Volmer laws, are presented in Table S3. All the oxidation reactions at the electrode are sufficiently fast so that the oxidations proceed under diffusion control.

Table S3. Butler-Volmer equations for determining the rate constants of electrode reactions of electroactive species.

Name	Butler-Volmer Equation	Description
kI	$k_o \cdot exp((1\text{-}alpha) \cdot f_a \cdot (E_a - E^o))$	Forward constant for TPrA oxidation
kA	$k_o \cdot exp(-alpha \cdot f_a \cdot (E_a - E^o))$	Backward constant for TPrA oxidation
kX	$k_o \cdot exp((1\text{-}alpha) \cdot f_a \cdot (E_a - E^o_d))$	Forward constant for $TPrA^{\bullet}$ oxidation
$kG_{2\to3}$	$k_{oRu} \cdot exp((1\text{-}alpha) \cdot f_a \cdot (E_a - E^o_{Ru}))$	Forward constant for $[Ru(bpy)_3]^{2+}$ oxidation
$kG_{3\to2}$	$k_{oRu} \cdot exp(-alpha \cdot f_a \cdot (E_a - E^o_{Ru}))$	Backward constant for $[Ru(bpy)_3]^{2+}$ oxidation
$kG_{1\to2}$	$k_{oRu} \cdot exp((1\text{-}alpha) \cdot f_a \cdot (E_a - E^o_{Ru\_}))$	Forward constant for $[Ru(bpy)_3]^{+}$ oxidation
$kG_{2\to1}$	$k_{oRu} \cdot exp(-alpha \cdot f_a \cdot (E_a - E^o_{Ru\_}))$	Backward constant for $[Ru(bpy)_3]^{+}$ oxidation

The inward flux (J) at the electrode surface can be expressed as follows in Eqs. 15-19:

$$J_{TPrA} = -\ J_{TPrA^{\bullet+}} = kI \cdot [TPrA^{\bullet+}] - kA \cdot [TPrA]$$
(15)

$$J_{TPrA^{\bullet}} = -\, kE \cdot [TPrA^{\bullet}]$$
(16)

$$J_{Ru^{3+}} = kG_{2\to3} \cdot [Ru^{2+}] - kG_{3\to2} \cdot [Ru^{3+}]$$
(17)

$$J_{Ru^{+}} = kG_{2\to1} \cdot [Ru^{2+}] - kG_{1\to2} \cdot [Ru^{+}]$$
(18)

$$J_{Ru^{2+}} = -\ J_{Ru^{3+}} - J_{Ru^{3+}}$$
(19)

where [TPrA], [$TPrA^{\bullet+}$], [$TPrA^{\bullet}$], [Ru^{2+}], [Ru^{3+}] and [Ru^{+}] represent the concentrations of TPrA, $TPrA^{\bullet+}$, $TPrA^{\bullet}$, $[Ru(bpy)_3]^{2+}$, $[Ru(bpy)_3]^{3+}$, and $[Ru(bpy)_3]^{+}$, respectively.

On the other hand, J at the bead surface was set in such a way to release $[Ru(bpy)_3]^{2+}$ upon consumption of $TPrA^{\bullet}$, as presented in Eq. 20:

$$J_{Ru^{2+}} = -J_{TPrA^{\bullet}} = kSS \cdot [TPrA^{\bullet}] \cdot \left[Ru^{2+}{}_{(bound)}\right]$$
(20)

where [$Ru^{2+}_{(bound)}$] and [$TPrA^{\bullet}$] represent the concentrations of $[Ru(bpy)_3]^{2+}$ on the bead surface and $TPrA^{\bullet}$, respectively.

The coverage of $[Ru(bpy)_3]^{2+}$ on the bead surface is described by the density of available sites ($3.8 \cdot 10^{-5}$ mol/m^2), intended here as the maximum number of COOH available for conjugation, and the initial concentration of Ru(II) ($2.1 \cdot 10^{-5}$ mol/m^2) roughly estimated considering a 55% conjugation efficiency. While release in solution of $[Ru(bpy)_3]^{2+}$ is described in “Bead Flux”, the consumption of the luminophore on the surface is described as a negative reaction rate in “Surface Reactions” as presented in Tab. S4.

The upper boundary in the model (Bulk in Fig. S8a) represent the bulk solution where the initial concentrations remain constant throughout the simulation, promoting diffusion along the z-axis. Isotropic

diffusion of species in solution is formulated according to the Fick's second law (Eq. 21), assuming that the contribution from the migration and convection is negligible:

$$\frac{\partial c_i}{\partial t} = D_i \Delta^2 c_i + R_i$$
(21)

where c_i and D_i represent the local concentration and diffusion coefficient of ith species, respectively, while t is the time, Δ represents the Laplacian, and R_i is the reaction flux necessary to maintain equilibrium.
We approximated the diffusion coefficient for $TPrAH^+$, TPrA, and its radicals to be $5 \cdot 10^{-10}$ $m^2 \cdot s^{-1}$ (D); while a diffusion coefficient of $5.9 \cdot 10^{-10}$ $m^2 \cdot s^{-1}$ is considered for $[Ru(bpy)_3]^{2+}$, its redox derivatives and its excited state (D_{Ru}). A faster diffusion coefficient of $9.3 \cdot 10^{-9}$ $m^2 \cdot s^{-1}$ was assigned to the proton H^+ (D_H) and, eventually, the slowest coefficient of $5 \cdot 10^{-54}$ $m^2 \cdot s^{-1}$ was attributed to hν (D_{min}).
The concentration change of every species involved in the ECL process as a consequence of Eqs. 5-14 are described in Table S4.

Table S4. Concentration changes of every species involved in the ECL process.

Concentration change	Expression	
$\frac{\partial[TPrAH^+]}{\partial t}$	$k_1 \cdot [TPrA] \cdot [H^+] - k_{1\_} \cdot [TPrAH^+]$	22
$\frac{\partial[TPrA]}{\partial t}$	$k_{1\_} \cdot [TPrAH^+] - k_1 \cdot [TPrA] \cdot [H^+] + k_{EI} \cdot [TPrA^{\bullet +}] \cdot [TPrA^{\bullet}] + k_{IG1} \cdot [Ru^+] \cdot [TPrA^{\bullet +}] - k_{AG3} \cdot [Ru^{3+}] \cdot [TPrA]$	23
$\frac{\partial[TPrA^{\bullet +}]}{\partial t}$	$k_3 \cdot [TPrA^{\bullet}] \cdot [H^+] - k_{3\_} \cdot [TPrA^{\bullet +}] - k_{EI} \cdot [TPrA^{\bullet +}] \cdot [TPrA^{\bullet}] - k_{IG1} \cdot [Ru^+] \cdot [TPrA^{\bullet +}] + k_{AG3} \cdot [Ru^{3+}] \cdot [TPrA]$	24
$\frac{\partial[TPrA^{\bullet}]}{\partial t}$	$k_{3\_} \cdot [TPrA^{\bullet +}] - k_3 \cdot [TPrA^{\bullet}] \cdot [H^+] - k_{EI} \cdot [TPrA^{\bullet +}] \cdot [TPrA^{\bullet}] - k_{EG} \cdot [Ru^{2+}] \cdot [TPrA^{\bullet}] - k_{EG3} \cdot [Ru^{3+}] \cdot [TPrA^{\bullet}]$	25
$\frac{\partial[H^+]}{\partial t}$	$k_{1\_} \cdot [TPrAH^+] - k_1 \cdot [TPrA] \cdot [H^+] + k_{3\_} \cdot [TPrA^{\bullet +}] - k_3 \cdot [TPrA^{\bullet}] \cdot [H^+]$	26
$\frac{\partial[Ru^{2+}{}_{(bound)}]}{\partial t}$	$-k_{SS} \cdot [TPrA^{\bullet}] \cdot [Ru^{2+}{}_{(bound)}]$	27
$\frac{\partial[Ru^{2+}]}{\partial t}$	$k_{AG3} \cdot [Ru^{3+}] \cdot [TPrA] + k_{G1G3} \cdot [Ru^{3+}] \cdot [Ru^+] + k_{G1} \cdot [Ru^+] + k_{em} \cdot [Ru^{2+*}] - k_{EG} \cdot [Ru^{2+}] \cdot [TPrA^{\bullet}] + k_{G1} \cdot [Ru^+]$	28
$\frac{\partial[Ru^{3+}]}{\partial t}$	$- k_{EG3} \cdot [Ru^{3+}] \cdot [TPrA^{\bullet}] - k_{AG3} \cdot [Ru^{3+}] \cdot [TPrA] - k_{G1G3} \cdot [Ru^{3+}] \cdot [Ru^+]$	29
$\frac{\partial[Ru^+]}{\partial t}$	$k_{EG} \cdot [Ru^{2+}] \cdot [TPrA^{\bullet}] - k_{IG1} \cdot [Ru^+] \cdot [TPrA^{\bullet +}] - k_{G1G3} \cdot [Ru^{3+}] \cdot [Ru^+] - k_{G1} \cdot [Ru^+]$	30
$\frac{\partial[Ru^{2+*}]}{\partial t}$	$k_{EG3} \cdot [Ru^{3+}] \cdot [TPrA^{\bullet}] + k_{IG1} \cdot [Ru^+] \cdot [TPrA^{\bullet +}] + k_{G1G3} \cdot [Ru^{3+}] \cdot [Ru^+] - k_{em} \cdot [Ru^{2+*}]$	31
$\frac{\partial[h\nu]}{\partial t}$	$k_{em} \cdot [Ru^{2+*}]$	32

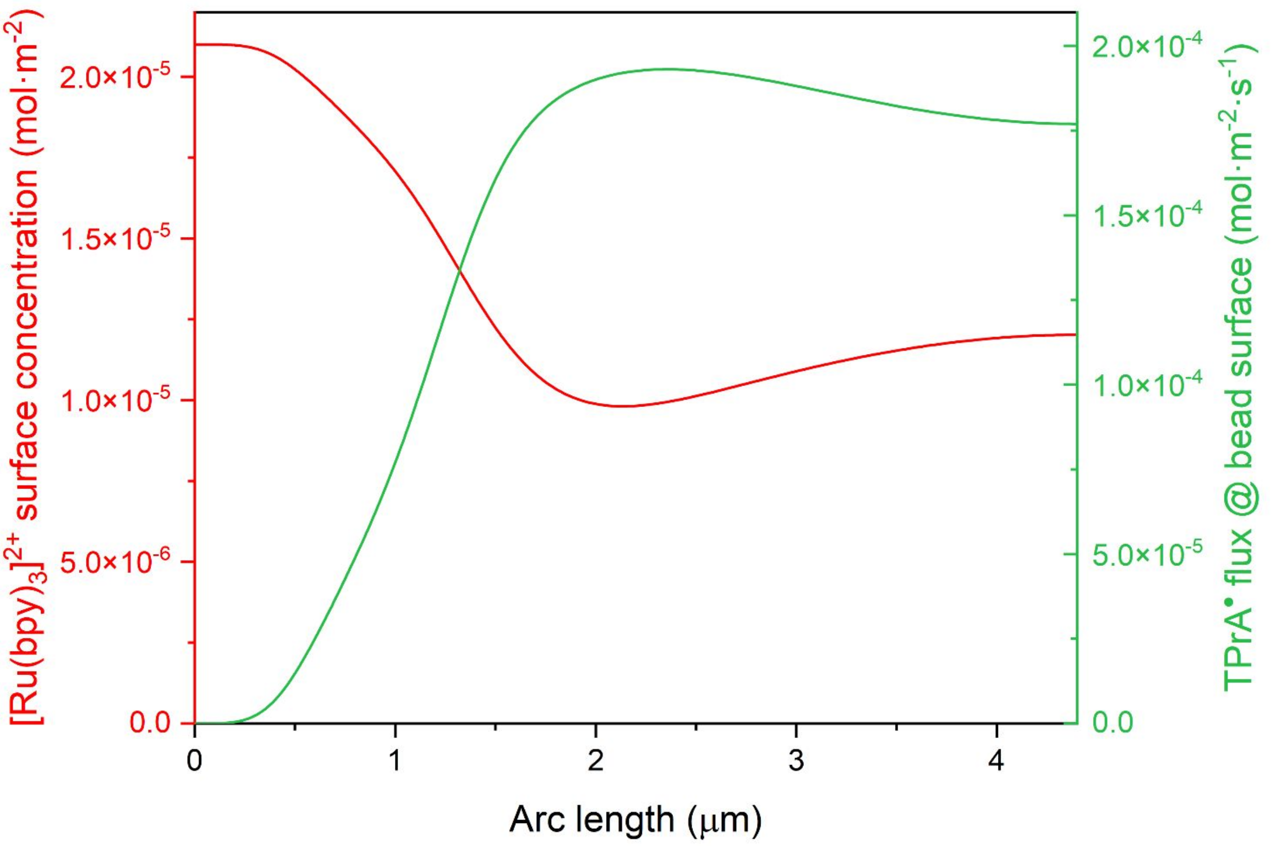

Figure S6. Dependence of the simulated concentration profiles of $[Ru(bpy)_3]^{2+}$ bounded to the bead (red line) and $TPrA^{\bullet}$ inward flux (green line) on the bead perimeter (where x = 0 µm represents the bead bottom and x = 4.4 µm represents the bead top).

ECL decay measurements

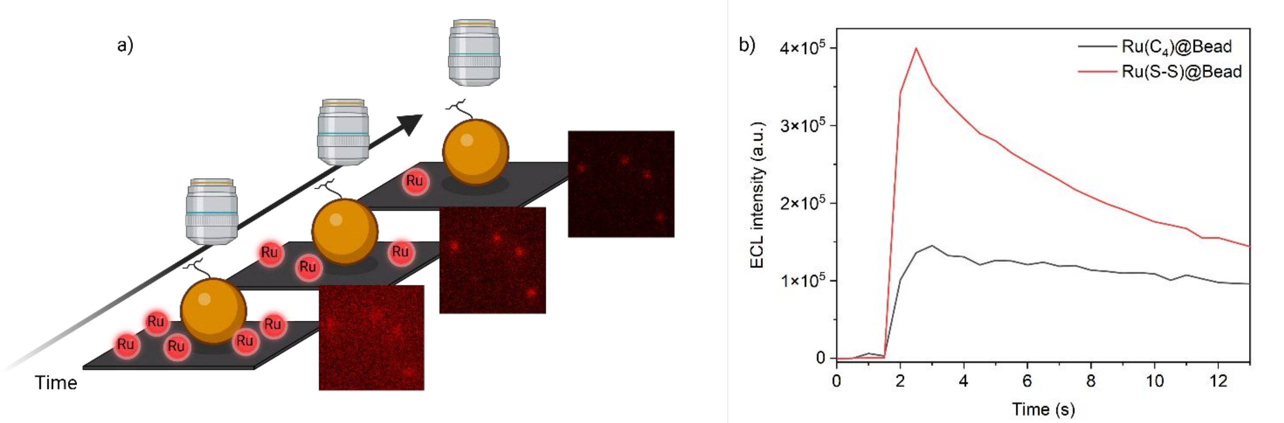

Figure S7. a) Schematic representation of monitoring ECL signal decay by collecting ECL images of labeled beads at 500 ms intervals during a two-step chronoamperometry . b) Comparison of single-bead ECL decay for Ru(C_4)@Bead (grey line) and Ru(S-S)@Bead (red line) in a 0.3 M PB solution with 180 mM TPrA (pH 6.8). Each data point represents a 500 ms frame captured during a two-step chronoamperometry measurement: 2 s at 0 V *vs* Ag/AgCl, followed 11 s at 2.5 V *vs* Ag/AgCl. For each frame, the ECL intensity was obtained by integrating the signal over a 7.94x7.94 µm ROI centered on the beads, subtracting the dark noise, and eventually averaging the results over a minimum of five beads (n ≥ 5).

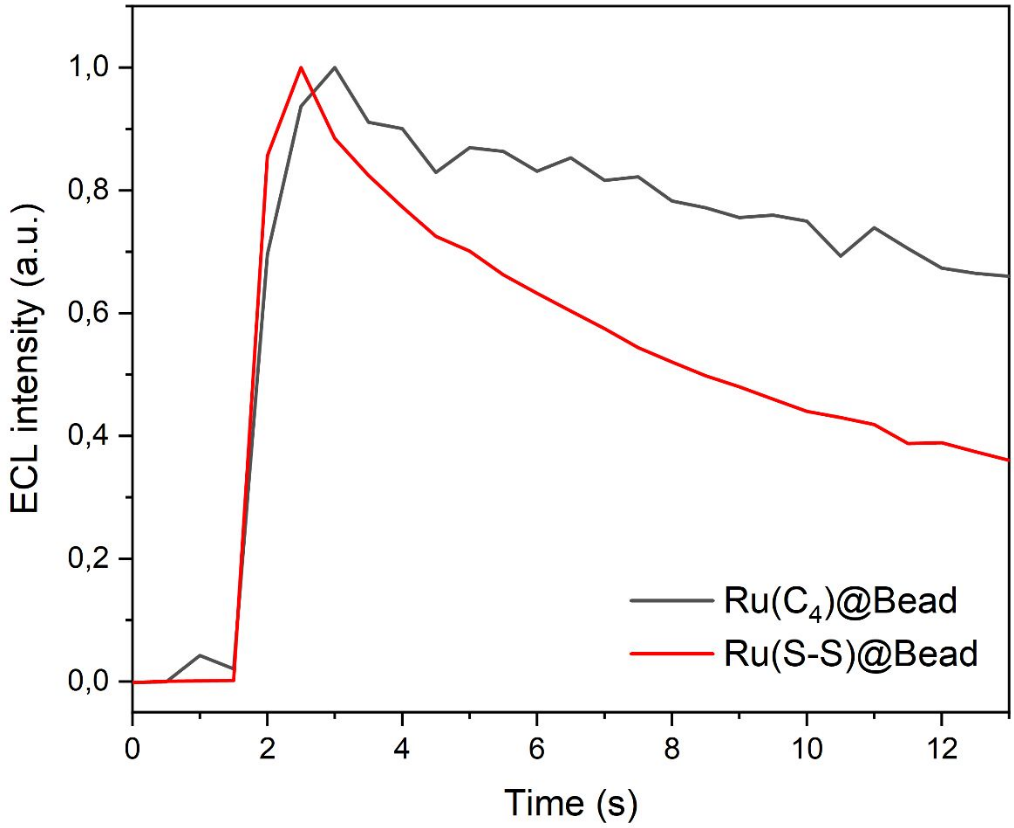

Figure S8. Comparison between normalized ECL decay of single beads for Ru(C_4)@Bead (grey line) and Ru(S-S)@Bead (red line), normalized from Fig. S5.

Diffusion of Ru(II) labels in solution

For each ECL imaging experiment, 1 µL of beads solution was deposited onto the working electrode surface. Based on the manufacturer data (up to 250 $pmol_{COOH}/\mu g_{beads}$), assuming a 100% functionalization yield (250 $pmol_{Ru(II)}/\mu g_{beads}$), and considering the concentration of the Ru(II)-labeled beads solution (0.72 $\mu g_{beads}/\mu L$), the amount of $[Ru(bpy)_3]^{2+}$ per ECL measurement is approximately 180 pmol. Given that the electrochemical cell used for ECL imaging has a capacity of 5 mL, the final concentration of Ru(II) complex released in solution would be 30 nM. However, it is important to note that 30 nM represents the best case scenario, as it assumes i) the highest possible density of COOH groups per bead, ii) a coupling yield of 100%, and that iii) all the $[Ru(bpy)_3]^{2+}$ units are released from the beads surface. The actual Ru(II) concentration in solution after equilibration is likely much smaller than theoretically predicted.

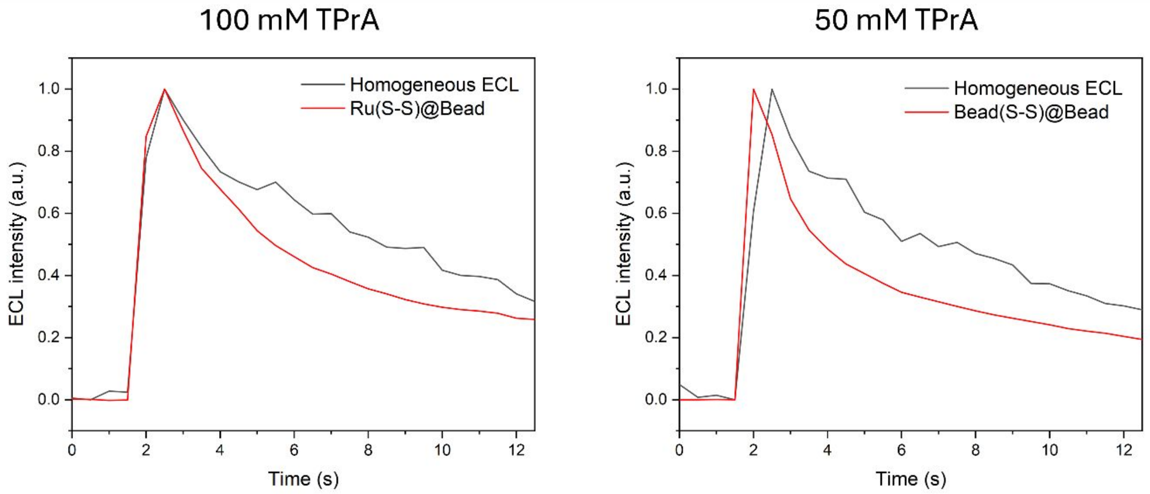

Figure S9. Comparison between normalized ECL decays of freely diffusing $[Ru(bpy)_3]^{2+}$ (25 nM, grey lines) and Ru(S-S)@Bead (red lines) with either 100 mM (left) or 50 mM (right) of TPrA.

ECL imaging at different TPrA concentrations on a GC electrode

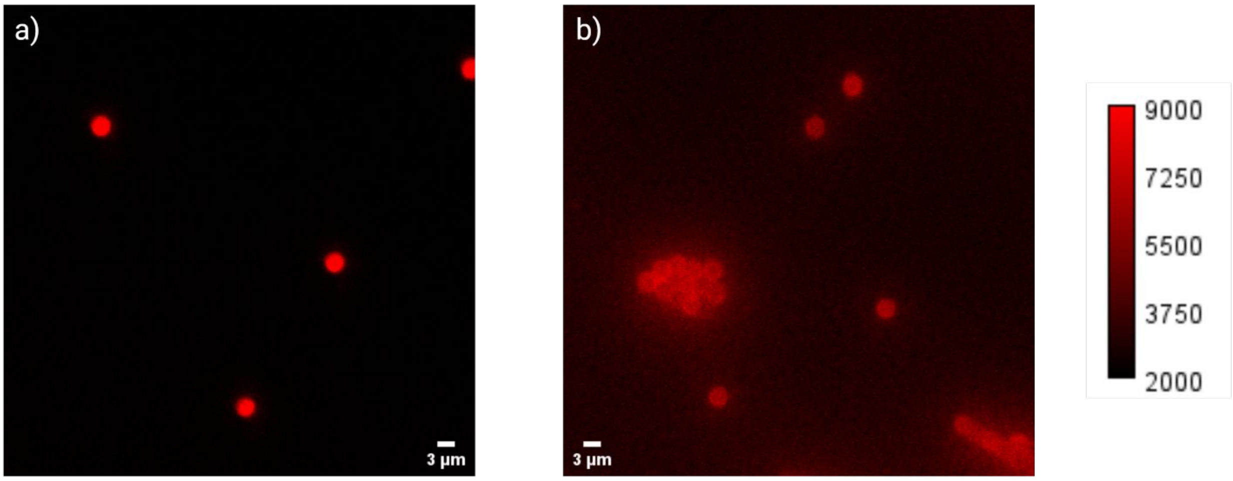

Figure S10. ECL images of a) Ru(C_4)@Bead and b) Ru(S-S)@Bead in 0.3 M PB with 180 mM TPrA (pH 6.8). The images were captured with an EM-CCD camera by recording the ECL signal for 13 s during a two-step chronoamperometry measurement: 2 s at 0 V *vs* Ag/AgCl and 11 s at 2.5 V *vs* Ag/AgCl. Magnification, X100; objective numerical aperture, 1.1; gain, 1; sensitivity, 250; contrast scale, 2000-9000; scale bar, 3 µm. A red lookup table was applied to the native greyscale images in a) and b) to generate false-color images resembling the emission of $[Ru(bpy)_3]^{2+}$.

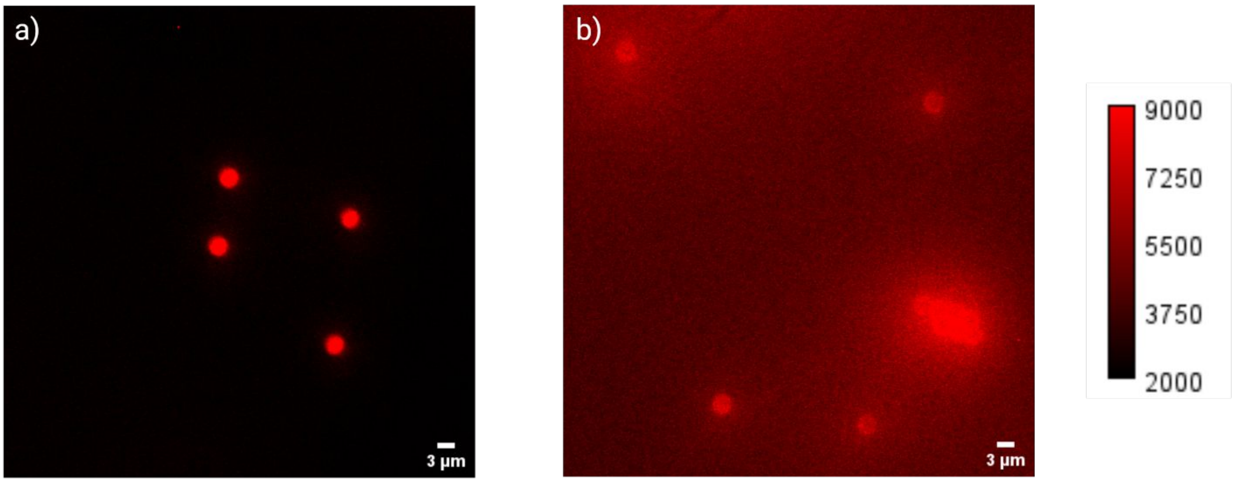

Figure S11. ECL images of a) Ru(C_4)@Bead and b) Ru(S-S)@Bead in 0.3 M PB with 100 mM TPrA (pH 6.8). The images were captured with an EM-CCD camera by recording the ECL signal for 13 s during a two-step chronoamperometry measurement: 2 s at 0 V *vs* Ag/AgCl and 11 s at 2.5 V *vs* Ag/AgCl. Magnification, X100; objective numerical aperture, 1.1; gain, 1; sensitivity, 250; contrast scale, 2000-9000; scale bar, 3 µm. A red lookup table was applied to the native greyscale images in a) and b) to generate false-color images resembling the emission of $[Ru(bpy)_3]^{2+}$.

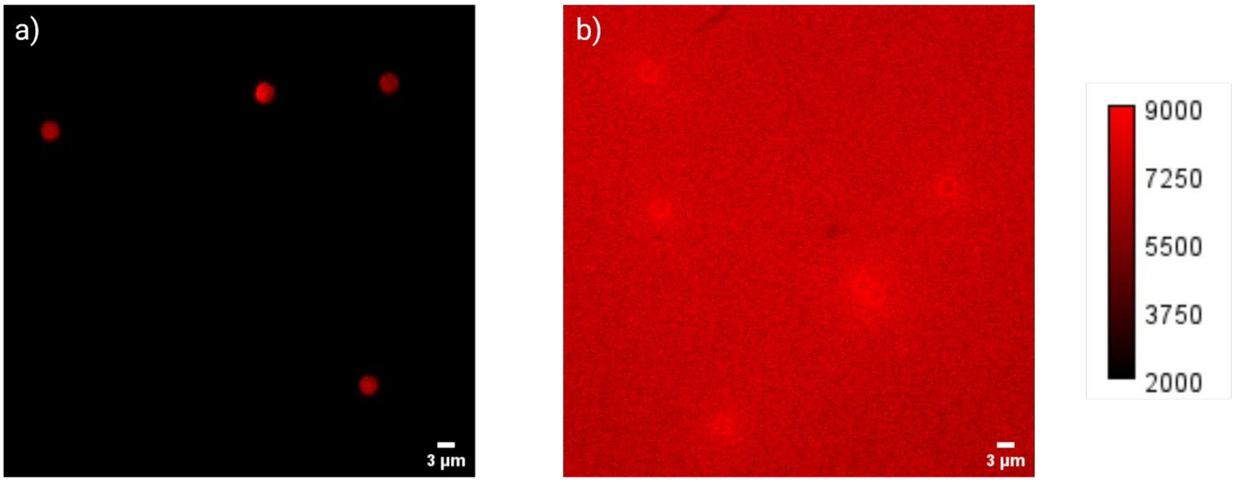

Figure S12. ECL images of a) Ru(C_4)@Bead and b) Ru(S-S)@Bead in 0.3 M PB with 50 mM TPrA (pH 6.8). The images were captured with an EM-CCD camera by recording the ECL signal for 13 s during a two-step chronoamperometry measurement: 2 s at 0 V *vs* Ag/AgCl and 11 s at 2.5 V *vs* Ag/AgCl. Magnification, X100; objective numerical aperture, 1.1; gain, 1; sensitivity, 250; contrast scale, 2000-9000; scale bar, 3 µm. A red lookup table was applied to the native greyscale images in a) and b) to generate false-color images resembling the emission of $[Ru(bpy)_3]^{2+}$.

ECL imaging at different TPrA concentrations on a Pt electrode

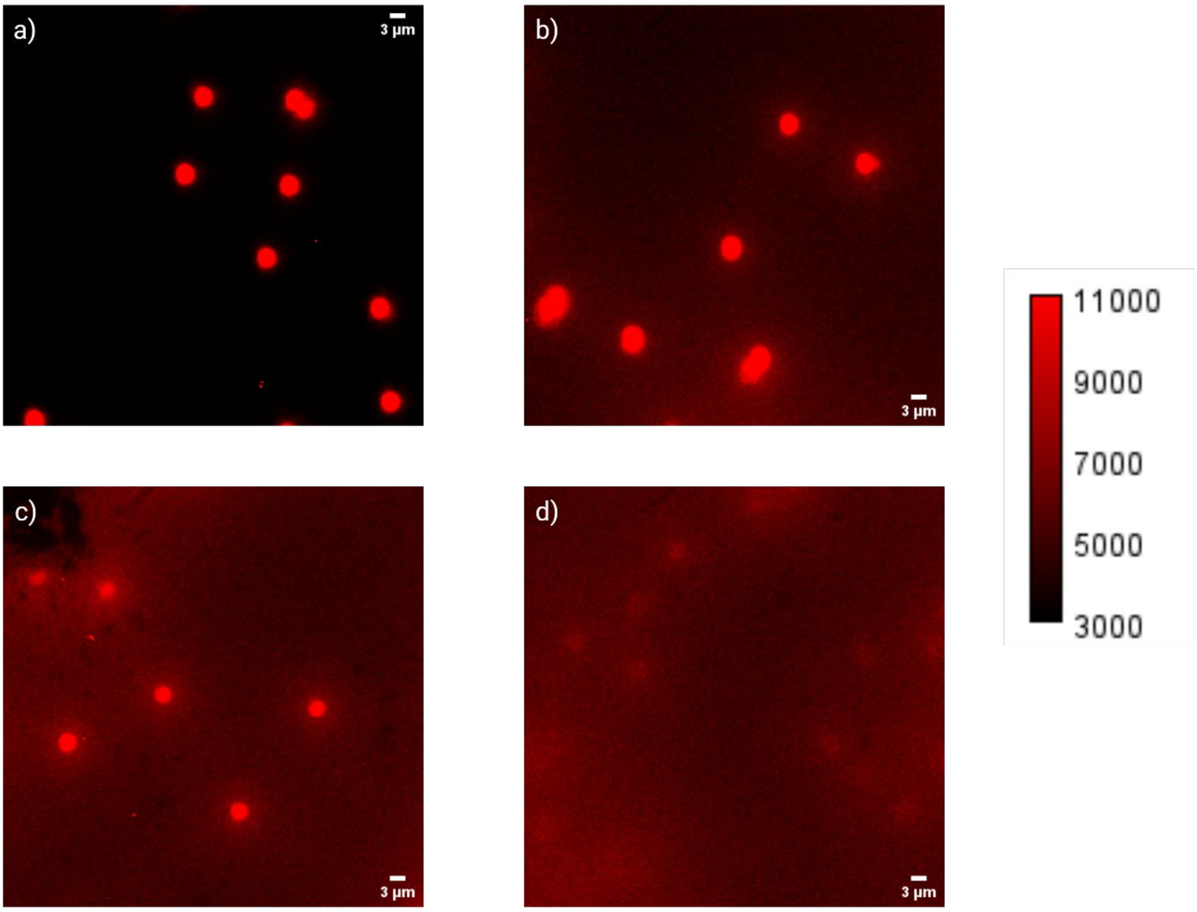

Figure S13. ECL images of a) Ru(C_4)@Bead and b) Ru(S-S)@Bead in 0.3 M PB with 180 mM TPrA (pH 6.8); c) Ru(S-S)@Bead in 0.3 M PB with 100 mM TPrA (pH 6.8); d) Ru(S-S)@Bead in 0.3 M PB with 50 mM TPrA (pH 6.8). The images were captured with an EM-CCD camera by recording the ECL signal for 45 s during a two-step chronoamperometry measurement: 2 s at 0 V *vs* Ag/AgCl and 43 s at 2.5 V *vs* Ag/AgCl. Magnification, X100; objective numerical aperture, 1.1; gain, 1; sensitivity, 250; contrast scale, 2000-9000; scale bar, 3 µm. A red lookup table was applied to the native greyscale images in a), b), c), and d) to generate false-color images resembling the emission of $[Ru(bpy)_3]^{2+}$.

ECL-TPrA concentration plot on Pt

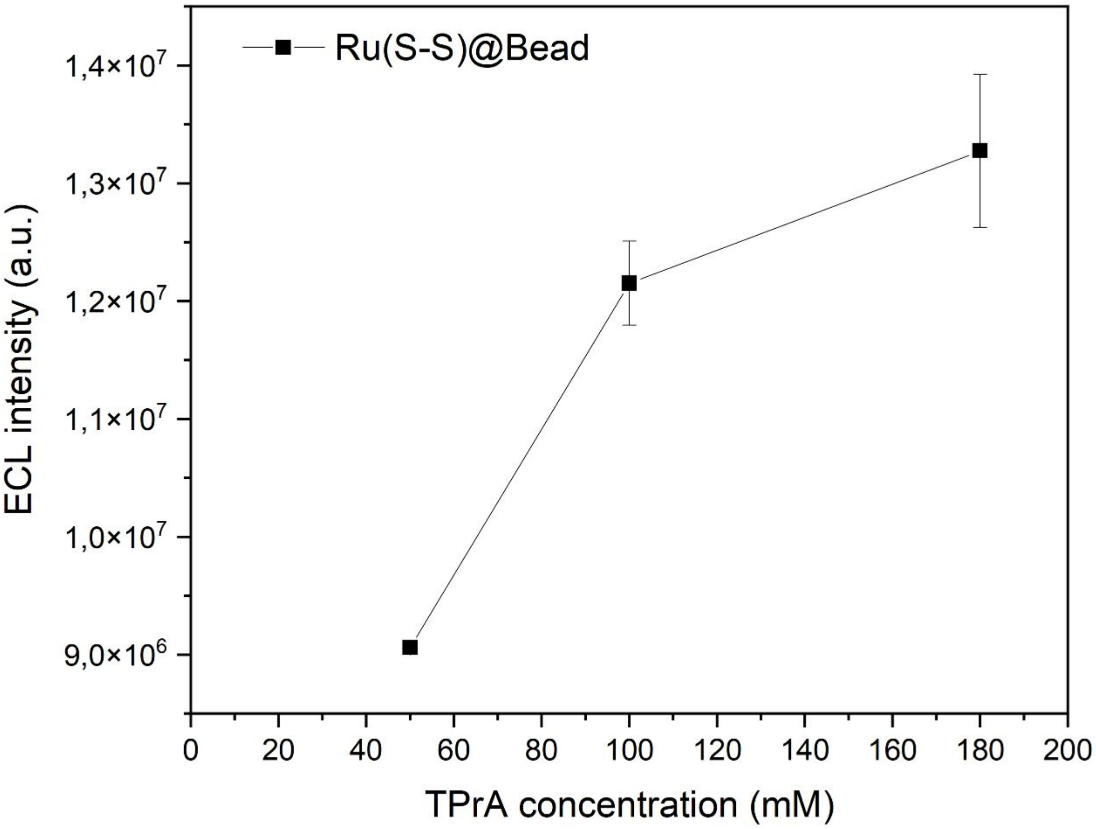

Figure S14. Effect of TPrA concentration on the ECL intensity of Ru(C_4)@Bead (grey line) and Ru(S-S)@Bead (red line) on a GC electrode. ECL intensities were determined from integrated ECL images, by integrating the signal over a ROI centered on the beads and averaging the results over a minimum of five beads ($n \geq 5$). Error bars represent the standard error. Interestingly, the behavior of Ru(S-S)@Bead on a Pt electrode is reversed compared to that on a GC surface, as the ECL intensity of labeled beads diminishes upon decreasing TPrA concentration. This trend was not unexpected as it is consistent with platinum being one of the least efficient materials for oxidizing TPrA. A lower concentration of coreactant further exacerbates the already slow oxidation rate, resulting in fewer radicals available to initiate the ECL process.

Ru(S-S)@Bead ECL at different TPrA concentrations on GC

ECL profiles

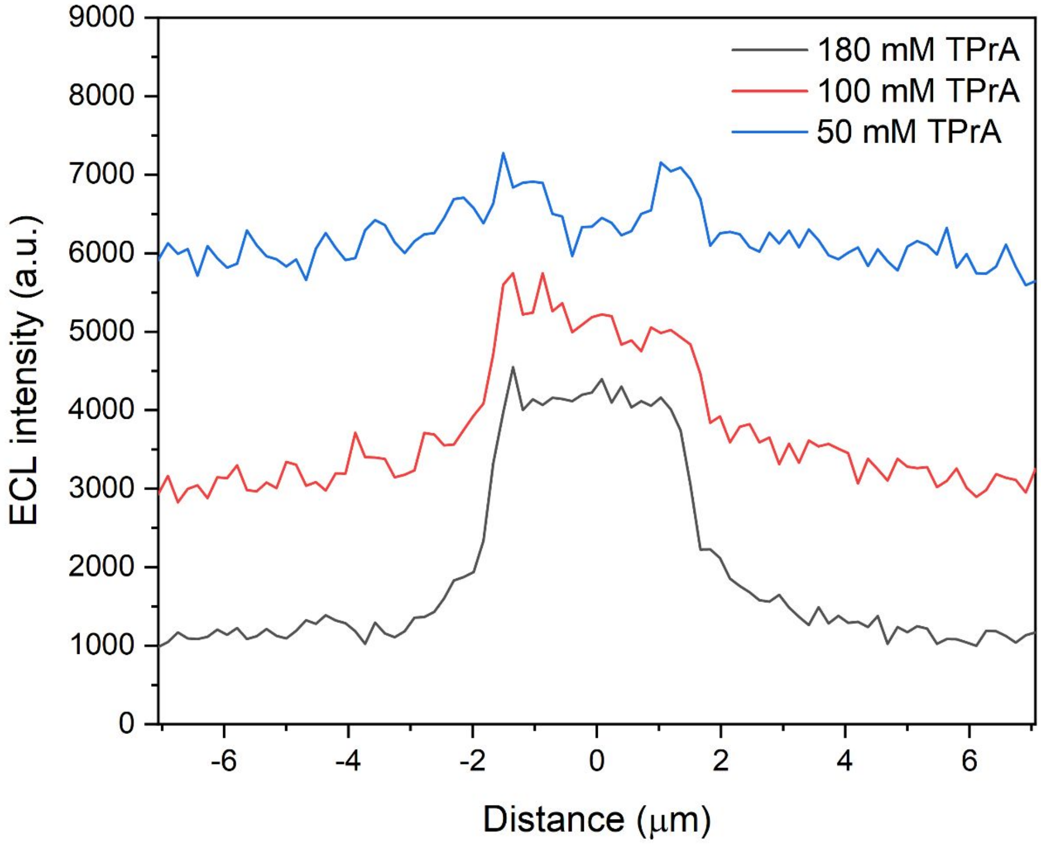

Figure S15. Single-bead ECL intensity profiles of Ru(S-S)@Bead on GC in 0.3 M PB with 50 mM (blue line), 100 mM (red line), and 180 mM (grey line) (pH 6.8). Data are averaged over a minimum of five beads ($n \geq 5$).

ECL-TPrA concentration plot on GC

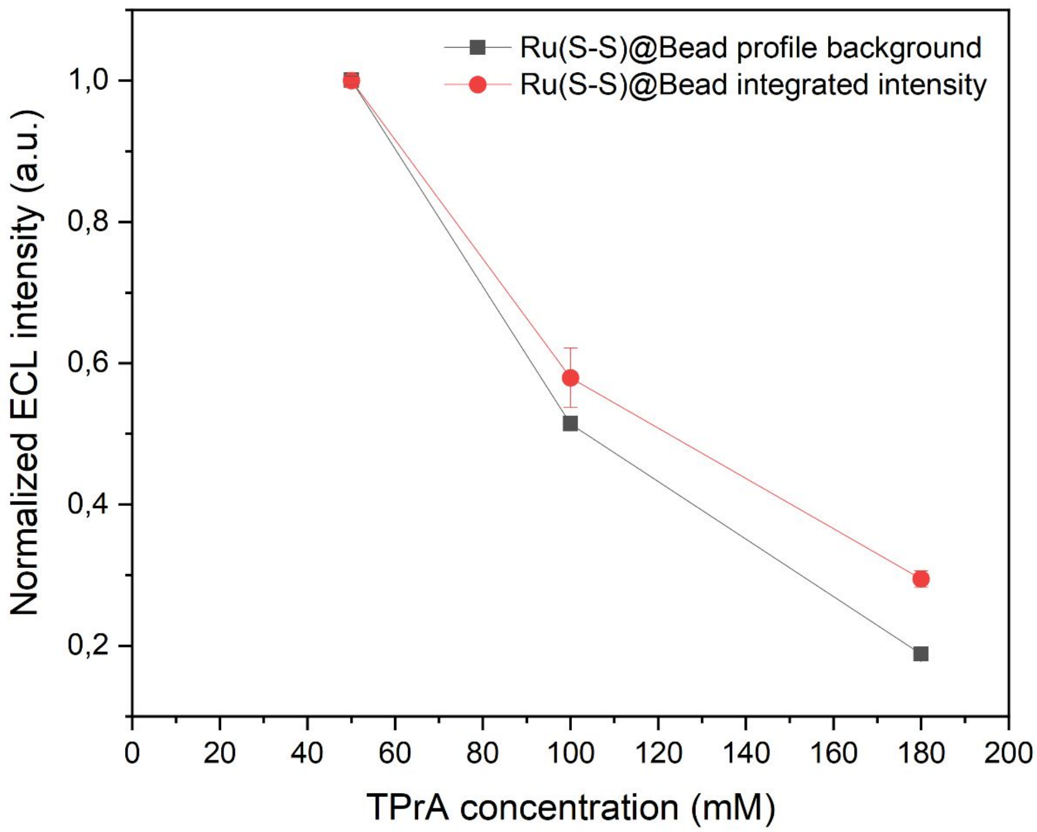

Figure S16. Effect of TPrA concentration on the normalized ECL intensity of Ru(S-S)@Bead including the emission from both the bead and the background (red line) and only the emission from the background (grey line) on a GC electrode. Integrated ECL intensities were determined from integrated ECL images, by integrating the signal over a ROI centered on the beads and averaging the results over a minimum of five beads ($n \geq 5$). Background ECL intensities were determined from the edges of the ECL profiles computed from integrated ECL images. Error bars represent the standard error.

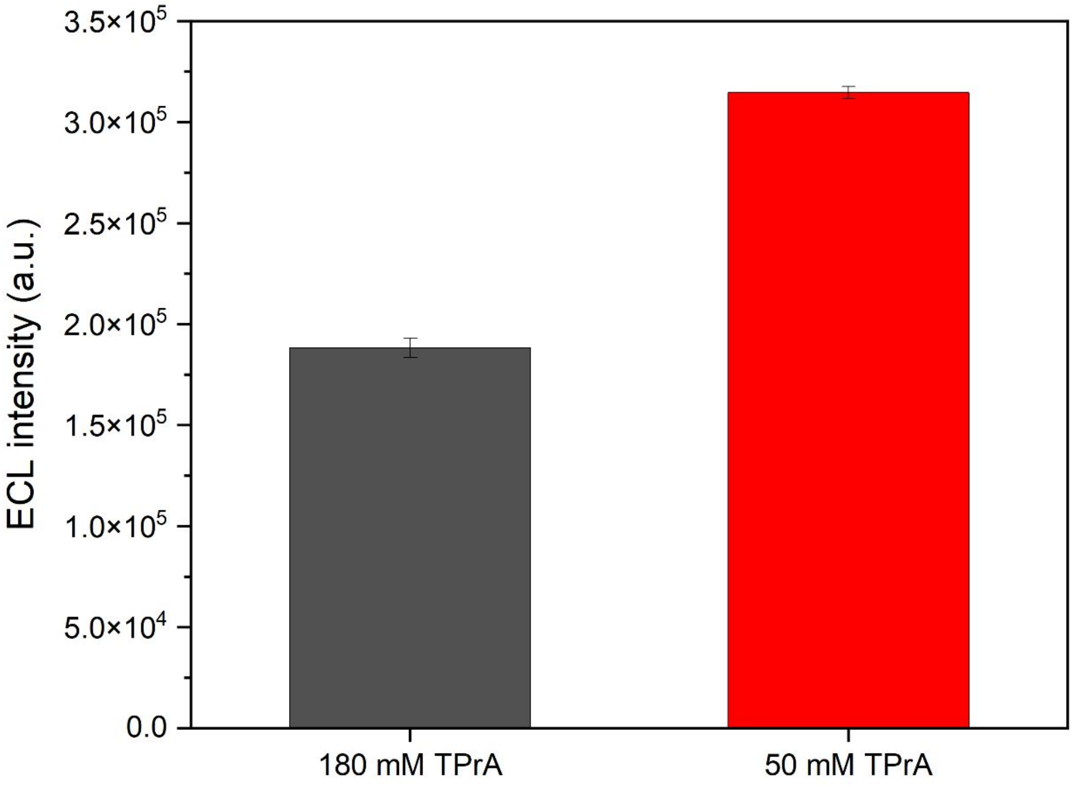

Figure S17. Comparison between ECL intensities of 25 nM $[Ru(bpy)_3]^{2+}$ in a 0.3 M PB solution with either 180 mM (grey bar) or 50 mM (red bar) of TPrA (pH 6.8). The ECL intensity was obtained from integrated ECL images over a 7.94x7.94 µm ROI.

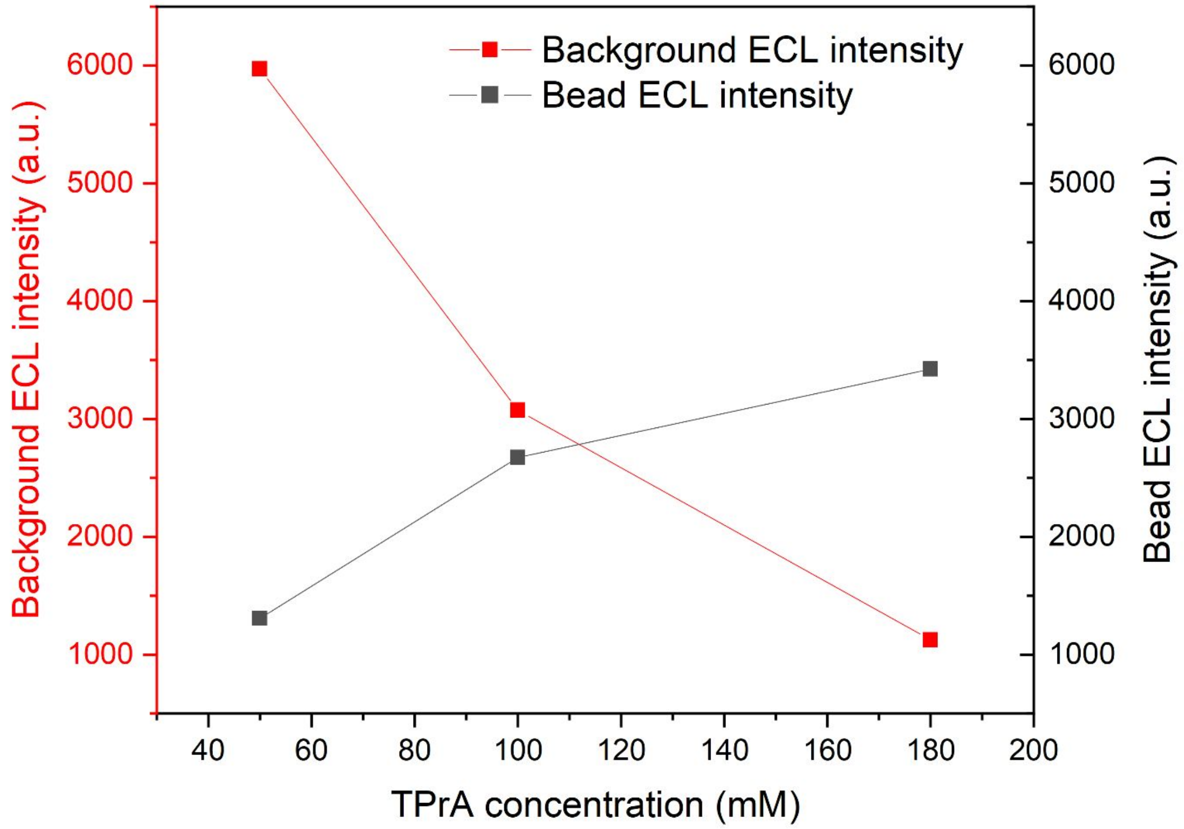

Figure S18. Effect of TPrA concentration on the ECL intensity on GC from Ru(II) labels on the beads surface (grey line) and from Ru(II) labels released in solution surrounding the beads (red line). The bead ECL intensity was determined from the difference between the ECL profile peak and the ECL intensity at the profiles edge, while the background ECL signal was considered as the intensity at the profiles edge.

Collective beads ECL

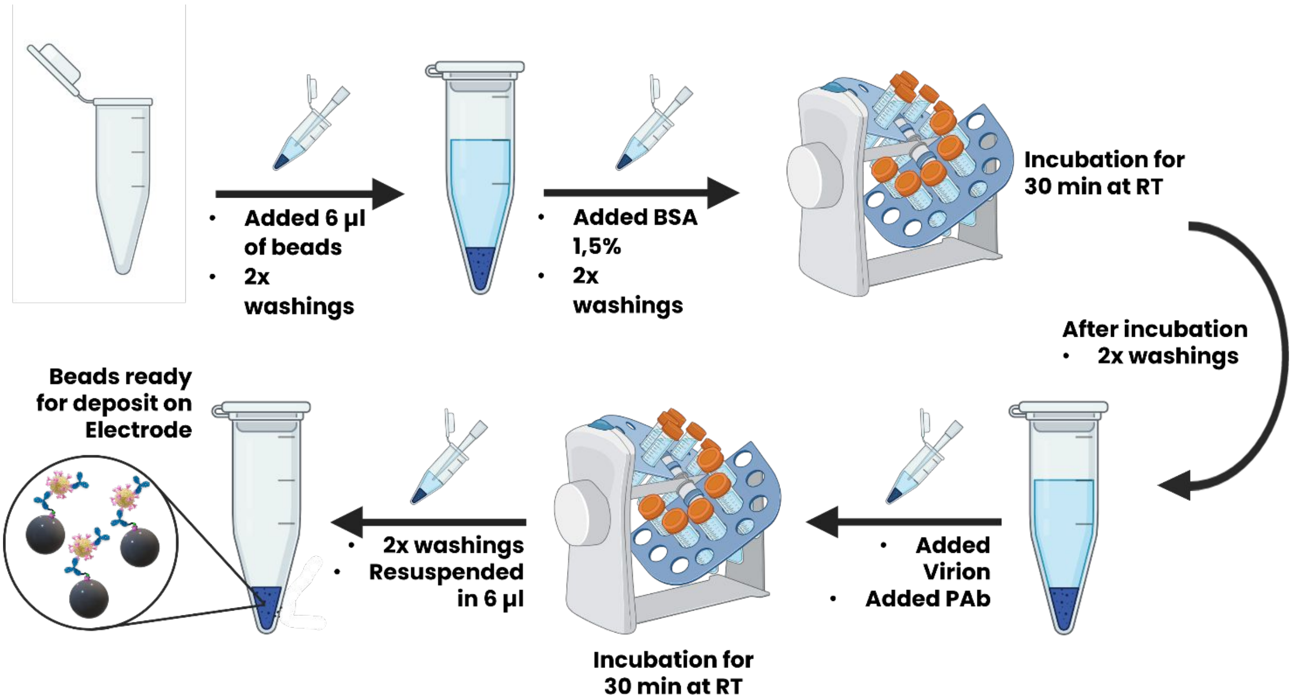

Figure S19. Preparation protocol of Ru(C_{27})@Ab-S1-Bead and Ru(S-S)(C_{21})@Ab-S1-Bead.

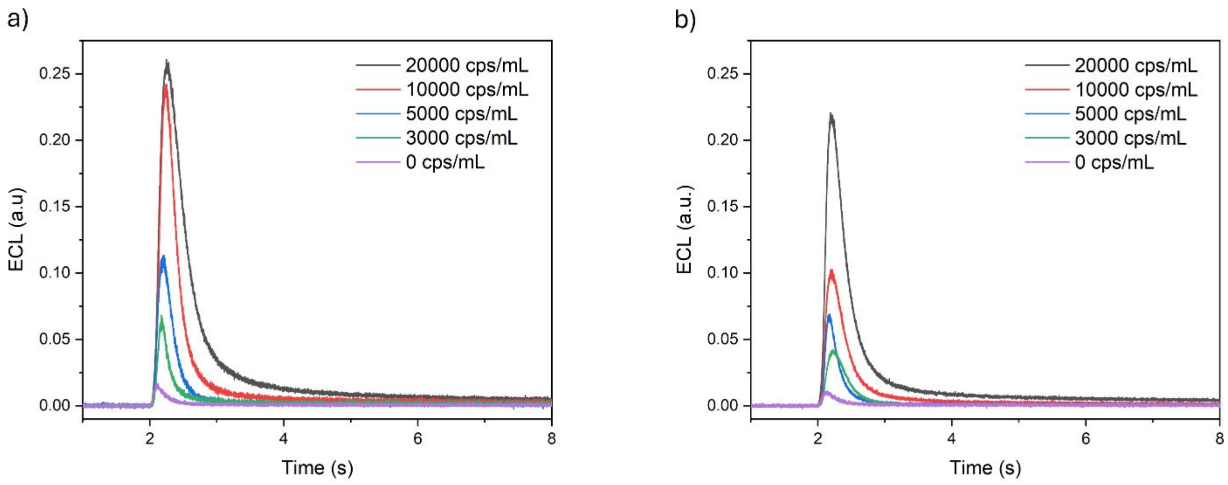

Figure S20. *ECL-time* curve of a) Ru(C_{27})@Ab-S1-Bead and b) Ru(S-S)(C_{21})@Ab-S1-Bead at different viral loadings on carbon screen printed electrodes in 0.3 M PB with 50 mM TPrA (pH 6.8). The ECL signal was collected with a PMT for 13 s during a two-step chronoamperometry measurement: 2 s at open circuit potential and 11 s at 1.9 V vs Ag/AgCl. PMT voltage, 0.750 V; amplification, 00.0 µA.

Literature overview for SARS-CoV-2 detection

Biosensor	Method	Analyte	Sample	LOD	Time	Ref.
**Microbeads-based immunoassay**	Voltammetric detection	S and N proteins	Saliva	19 ng/mL and 8 ng/mL respectively, for S and N protein	2 h	Biosens. Bioelectron. 2021, 171, 112686[4]
**Electro-immunosorbent assay**	Impedimetric detection	N protein	PBS	0.1 ng/mL	5 h	ACS Sens. 2022, 7, 1676[5]
**Plasmonic sensor**	Colorimetric detection	S protein	Saliva and river water	0.28 PFU/mL	15 h	ACS Appl. Mater. Interfaces 2022, 14, 54527[6]
**Microbeads-based immunoassay**	Colorimetric detection	S protein	Saliva	0.1 µg/mL	2 h	Biosens. Bioelectron. 2022, 200, 113909[7]
**Lateral flow immunoassay**	Fluorescence detection	S and N proteins	Saliva and swab	0.5 pg/mL	5 h	ACS Appl. Mater. Interfaces 2021, 13, 40342[8]
**Immunoassay**	ECL detection	N protein	Serum	3 pg/mL	16 h	Anal. Chem. 2024, 96, 17345[9]
**Lateral flow immunoassay**	ECL detection	N protein	Buffer	0.52 ng/mL	> 24 h	Adv. Funct. Mat. 2024, 34, 2409632[10]
**Microbeads-based immunoassay**	ECL detection	S protein	Swab	1103 cps/mL	50 min	This work

Table S1. Overview of biosensors for SARS-CoV-2 detection.

References

(1) Zeglis, B. M.; Barton, J. K. A Mismatch-Selective Bifunctional Rhodium-Oregon Green Conjugate: A Fluorescent Probe for Mismatched DNA. *J. Am. Chem. Soc.* **2006**, *128* (17), 5654–5655

(2) Fisher, K. J.; Turkett, J. A.; Corson, A. E.; Bicker, K. L. Peptoid Library Agar Diffusion (PLAD) Assay for the High-Throughput Identification of Antimicrobial Peptoids. *ACS Comb. Sci.* **2016**, *18* (6), 287–291

(3) Fan, Z.; Wu, J.; Liu, W.; Ma, J.; Sun, J.; Wang, P. Thiol-Selective Sensor Based on Intramolecular Energy Transfer between a Bichromophoric System. *Tetrahedron* **2013**, *69* (23), 4536–4540

(4) Fabiani, L.; Saroglia, M.; Galatà, G.; De Santis, R.; Fillo, S.; Luca, V.; Faggioni, G.; D'Amore, N.; Regalbuto, E.; Salvatori, P.; Terova, G.; Moscone, D.; Lista, F.; Arduini, F. Magnetic Beads Combined with Carbon Black-Based Screen-Printed Electrodes for COVID-19: A Reliable and Miniaturized Electrochemical Immunosensor for SARS-CoV-2 Detection in Saliva. *Biosens. Bioelectron.* **2021**, *171*, 112686

(5) Cho, H.; Shim, S.; Cho, W. W.; Cho, S.; Baek, H.; Lee, S. M.; Shin, D. S. Electrochemical Impedance-Based Biosensors for the Label-Free Detection of the Nucleocapsid Protein from SARS-CoV-2. *ACS Sensors* **2022**, *7* (6), 1676–1684

(6) Materón, E. M.; Gómez, F. R.; Almeida, M. B.; Shimizu, F. M.; Wong, A.; Teodoro, K. B. R.; Silva, F. S. R.; Lima, M. J. A.; Angelim, M. K. S. C.; Melendez, M. E.; Porras, N.; Vieira, P. M.; Correa, D. S.; Carrilho, E.; Oliveira, O. N.; Azevedo, R. B.; Goncalves, D. Colorimetric Detection of SARS-CoV-2 Using Plasmonic Biosensors and Smartphones. *ACS Appl. Mater. Interfaces* **2022**, *14* (49), 54527–54538

(7) Fabiani, L.; Mazzaracchio, V.; Moscone, D.; Fillo, S.; De Santis, R.; Monte, A.; Amatore, D.; Lista, F.; Arduini, F. Paper-Based Immunoassay Based on 96-Well Wax-Printed Paper Plate Combined with Magnetic Beads and Colorimetric Smartphone-Assisted Measure for Reliable Detection of SARS-CoV-2 in Saliva. *Biosens. Bioelectron.* **2022**, *200*, 113909

(8) Wang, C.; Cheng, X.; Liu, L.; Zhang, X.; Yang, X.; Zheng, S.; Rong, Z.; Wang, S. Ultrasensitive and Simultaneous Detection of Two Specific SARS-CoV-2 Antigens in Human Specimens Using Direct/Enrichment Dual-Mode Fluorescence Lateral Flow Immunoassay. *ACS Appl. Mater. Interfaces* **2021**, *13* (34), 40342–40353

(9) Wu, P.; Zhang, L.; Zhang, G.; Cheng, L.; Zhang, F.; Li, Y.; Lei, Y.; Qi, H.; Zhang, C.; Gao, Q. Highly Sensitive Electrochemiluminescence Biosensing Method for SARS-CoV-2 N Protein Incorporating the Micelle Probes of Quantum Dots and Dibenzoyl Peroxide Using the Screen-Printed Carbon Electrode Modified with a Carboxyl-Functionalized Graphene. *Anal. Chem.* **2024**, *96* (43), 17345–17352

(10) Fu, W.; Wang, X.; Ying, X.; Sun, T.; Wang, Y.; Wang, J.; Su, B. Electrochemiluminescence Lateral Flow Immunoassay Using Ruthenium(II) Complex-Loaded Dendritic Mesoporous Silica Nanospheres for Highly Sensitive and Quantitative Detection of SARS-CoV-2 Nucleocapsid Protein. *Adv. Funct. Mater.* **2024**, *34*, 2409632
